# Theory of terahertz pump optical probe spectroscopy of phonon polaritons in noncentrosymmetric systems

**DOI:** 10.1038/s41535-025-00761-8

**Published:** 2025-05-01

**Authors:** Niccolò Sellati, Jacopo Fiore, Stefano Paolo Villani, Lara Benfatto, Mattia Udina

**Affiliations:** Department of Physics, Sapienza University of Rome, P.le A. Moro 5, 00185 Rome, Italy

**Keywords:** Condensed-matter physics, Nonlinear optics, Polaritons, Terahertz optics, Ultrafast photonics

## Abstract

Hybrid lattice-light modes, known as phonon polaritons, represent the backbone of advanced protocols based on THz pumping of infrared modes. Here we provide a theoretical framework able to capture the different roles played by phonon polaritons in experimental protocols based either on Raman-like pump and probe schemes, typical of four-wave mixing processes, or on THz pump-visible probe three-wave mixing protocols. By using a many-body description of the nonlinear optical kernel, along with a perturbative solution of nonlinear Maxwell’s equations, we discuss the limitations of all-optical four-wave mixing protocols and we highlight the advantages of exploiting broadband THz pumps to enlarge the phase space of the phonon polariton dispersion at low momenta accessible in a single experiment. Besides providing a quantitative description of existing and future experiments, our results offer a general framework for the theoretical modeling of the hybridization between light and lattice degrees of freedom in time-resolved experiments.

## Introduction

The interaction between infrared-active lattice vibrations and propagating electromagnetic (e.m.) fields leads to the emergence of hybrid light-phonon modes in solid state compounds, named phonon polaritons (PhPs)^1–8^. In noncentrosymmetric crystals IR-active phonons can also be Raman-active, and the PhPs can be investigated by means of coherent nonlinear spectroscopy as in four-wave mixing (FWM) or three-wave mixing (TWM) protocols. In the former both the excitation and detection mechanisms rely on a Raman-like coupling of the phonon mode to light, as is the case for Impulsive Stimulated Raman Scattering (ISRS)^3,9–18^, where the PhP dispersion is measured by tuning the frequency of a visible pulse and taking advantage of momentum conservation of phase-matched processes. A somehow analogous mechanism underlies older measurements based on nearly-forward spontaneous Raman scattering^19–21^, where the phase-matching condition was tuned by changing the angle between the incident and scattered light. Thanks to the huge technical improvements in the generation of intense THz light pulses^22,23^ the detection of PhPs is nowadays possible also via THz pump-optical probe TWM processes. In this case the IR excitation of the phonon can be achieved with a THz pulse, and the PhP can be detected as reflectivity or transmission changes of a time-delayed optical probe via a Raman-like interaction^22,24–30^. A similar TWM mechanism has been exploited in the past in Raman experiments, by simultaneously irradiating the sample with a continuous-wave IR laser^31–36^. In this configuration, the main observation is that PhPs with a finite momentum mismatch can be excited and detected, even though with a still unexplained marked modulation of the detection signal. So far, a complete theoretical characterization of the role played by the hybrid character of the PhP, the phase mismatch and the propagation effects in the different multiwave protocols has not been provided yet, hindering the potential applications of PhPs in phonon-driven phase transitions and in their interaction with additional collective modes^10,37–44^.

In this work we develop a general theoretical framework to describe the detection of PhPs in noncentrosymmetric cubic systems through FWM and TWM protocols. We combine a many-body derivation of the nonlinear current involved in multiwave mixing processes with a perturbative solution of Maxwell’s equations in the presence of a nonlinear source current, in order to correctly describe propagation effects related to the phase-matching conditions. We show that in the FWM case one directly accesses the phonon component of the PhP, explaining both stimulated Raman, such as ISRS^3,9–18^, as well as spontaneous Raman^19–21^ experiments. The formalism is then applied to TWM protocols based on THz pump-optical probe experiments with a narrowband (multi-cycle) or broadband (single-cycle) pump. We demonstrate that in this case the PhP dispersion enters mainly via the propagation effects of the nonlinear signal generated by the THz pump. For narrowband pulses, that closely resemble Raman experiments stimulated with continuous-wave IR radiation^31–36^, we explain how the signal modulation depends on the generation of PhPs with a finite momentum mismatch. For broadband pulses we show how transmission measurements can access the polariton dispersion by tuning the spectral features of the pump and probe pulses, making TWM a preferential knob to investigate the light-matter hybridization process.

## Results

As a starting point we model the IR-active phonon mode as an harmonic oscillator of frequency *ω*_TO_. Given the phonon displacement **Q**(*iΩ*_*m*_, **k**) and phonon momentum **P**(*i*Ω_*m*_, **k**), with Ω_*m*_ = 2*πm*/*β* the bosonic Matsubara frequencies and *β* the inverse temperature, one can write the Gaussian action in the path integral formulation^45^ as $S[{\bf{Q}},{\bf{P}}]=\sum _{i{\Omega }_{m},{\bf{k}}}\left[H(i{\Omega }_{m},{\bf{k}})+{\Omega }_{m}{\bf{P}}(i{\Omega }_{m},{\bf{k}})\cdot {\bf{Q}}(-i{\Omega }_{m},-{\bf{k}})\right]$, where $H(i{\Omega }_{m},{\bf{k}})={\omega }_{{\rm{TO}}}^{2}| {\bf{Q}}(i{\Omega }_{m},{\bf{k}}){| }^{2}/2+| {\bf{P}}(i{\Omega }_{m},{\bf{k}}){| }^{2}/2$ gives the phonon Hamiltonian. By integrating out **P**, one is left with ${S}_{0}[{\bf{Q}}]={\sum }_{i{\Omega }_{m},{\bf{k}}}[{D}_{0}^{-1}| {\bf{Q}}(i{\Omega }_{m},{\bf{k}}){| }^{2}]$, where ${D}_{0}(i{\Omega }_{m})=2/({\omega }_{{\rm{TO}}}^{2}+{\Omega }_{m}^{2})$ is the bare phonon propagator. To model multiwave detection processes, we must include all possible couplings between the phonon and the e.m. field. Since the mode is IR-active, the system admits a linear coupling to the gauge field **A** that can be included via the minimal coupling substitution^46^**P**(*iΩ*_*m*_, **k**) → **P**(*iΩ*_*m*_, **k**) − Z**A**(*iΩ*_*m*_, **k**)/*c* in *H*(*iΩ*_*m*_, **k**), where *c* is the speed of light and Z is a rank-2 tensor that can be connected to the Born effective charge. In cubic crystals Z is scalar, such that longitudinal and transverse components are decoupled. Integrating **P** one is left with ${S}_{{\rm{IR}}}[{\bf{Q}},{\bf{A}}]={\sum }_{i{\Omega }_{m},{\bf{k}}}[{\Omega }_{m}({\rm{Z}}{\bf{A}}/c)\cdot {\bf{Q}}]$. From now on we only focus on the coupling of the transverse components of the phonon **Q**_*T*_ to the e.m. field. Whenever the mode is also Raman-active one should add an additional quadratic (Raman-like) coupling term ${S}_{R}[{{\bf{Q}}}_{T},{\bf{A}}]={\sum }_{i{\Omega }_{m},{\bf{k}}}{\sum }_{i{\Omega }_{n},{{\bf{k}}}^{{\prime} }}[{{\bf{Q}}}_{T}{\mathcal{R}}{\bf{A}}{\bf{A}}]$^47^, where ${\mathcal{R}}$ is a rank-3 tensor that can be connected to the phonon Raman tensor. Finally, one should include the quantum action *S*_e.m._[**A**] describing the fluctuations of the e.m. field. In the presence of external perturbations some spectral components of **A** are connected to the pump or probe fields, and one is interested in computing a nonlinear current **J**^(n)^ (with *n* = 2 for TWM and *n* = 3 for FWM) with respect to them by integration of any other fluctuating components.

### Four-wave mixing interaction

**Fig. 1:**
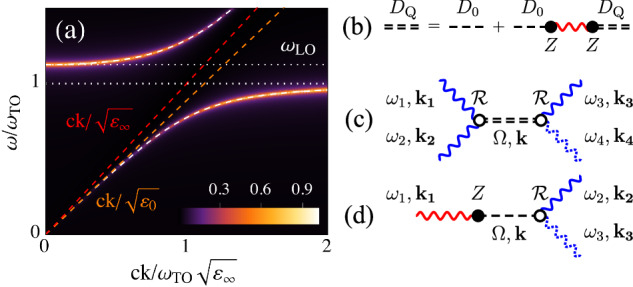
Phonon-mediated multiwave mixing processes. **a** Absolute value of the PhP spectral function, obtained from Eq. (1), normalized to its maximum value. Horizontal white dotted lines highlight the *ω*_TO_ and *ω*_LO_ frequencies (here *ω*_LO_ = 1.125*ω*_TO_). White dashed curves are the polaritonic dispersion branches. Here we set *γ* = 0.01*ω*_TO_. **b** Dyson equation for *D*_Q_ (double dashed line), obtained by integrating out the e.m. field (red line) to dress the bare phonon propagator *D*_0_ (single dashed line). **c** FWM interaction between three optical photons **A**_*R*_ (blue lines), mediated by the PhP. **d** TWM interaction between the THz field **A**_*p*_ (red line) and the optical field **A**_*R*_ (blue line), mediated by the bare phonon. The nonlinear current **J**^(n)^ related to the two processes is obtained as the functional derivative with respect to the outgoing optical field (dashed blue line).

To clarify the approach, let us start from FWM processes. In this case the pump and the probe are represented by a visible field **A**_*R*_. The THz field, linearly coupled to the phonon, can be integrated out as its spectral components are well separated from those of **A**_*R*_. Such procedure, detailed in Supplementary Note 1 and Supplementary Note 2, has the primary effect of dressing the bare phonon with the THz e.m. fields, leading to a PhP mode coupled to the external perturbation **A**_*R*_, *S*[**Q**_*T*_, **A**_*R*_] = *S*_*G*_[**Q**_*T*_] + *S*_*R*_[**Q**_*T*_, **A**_*R*_], where ${S}_{G}[{{\bf{Q}}}_{T}]={\sum }_{i{\Omega }_{m},{\bf{k}}}[{D}_{{\rm{Q}}}^{-1}{\left\vert {{\bf{Q}}}_{T}(i{\Omega }_{m},{\bf{k}})\right\vert }^{2}]$ and

1 $${D}_{{\rm{Q}}}(i{\Omega }_{m},{\bf{k}})=\frac{2}{{\omega }_{{\rm{TO}}}^{2}+{\Omega }_{m}^{2}+\frac{{\Omega }_{P}^{2}{\Omega }_{m}^{2}}{{\Omega }_{m}^{2}+\frac{{c}^{2}}{{\varepsilon }_{\infty }}| {\bf{k}}{| }^{2}}}$$

is the propagator of the phonon dressed by the interaction with light, see Fig. 1a, b. In this expression *ε*_*∞*_ is the high-frequency dielectric constant and ${\Omega }_{P}^{2}$ is the ionic plasma frequency proportional to the matrix elements of Z^2^, entering the definition of the longitudinal-optical phonon frequency as ${\omega }_{{\rm{LO}}}^{2}={\omega }_{{\rm{TO}}}^{2}+{\Omega }_{P}^{2}$. Eq. (1) can equivalently be written as ${D}_{{\rm{Q}}}={D}_{0}({\varepsilon }_{\infty }{\Omega }_{m}^{2}+{c}^{2}| {\bf{k}}{| }^{2})/(\varepsilon (i{\Omega }_{m}){\Omega }_{m}^{2}+{c}^{2}| {\bf{k}}{| }^{2})$ where the dielectric function reads $\varepsilon (i{\Omega }_{m})={\varepsilon }_{\infty }\left({\Omega }_{m}^{2}+{\omega }_{{\rm{LO}}}^{2}\right)/\left({\Omega }_{m}^{2}+{\omega }_{{\rm{TO}}}^{2}\right)$. Once the analytical continuation to real frequencies *iΩ*_*m*_ → *ω* + *i*0^+^ is performed, the poles of the propagator identify the dispersion of the phonon mode. One then recovers the two polaritonic branches, given by the wave equation *ε*(*ω*)*ω*^2^ = *c*^2^∣**k**∣^2^^46^, weighted by the factor *ε*_*∞*_*ω*^2^ − *c*^2^∣**k**∣^2^ that accounts for the phonon character of the polariton. This weight affects the intensity of the phonon spectral function *G*_Q_(*ω*, **k**) = − Im[*D*_Q_(*i*Ω_*m*_ → *ω* + *iγ*, **k**)]/*π* shown in Fig. 1a, where we introduced a finite imaginary part *i*0^+^ → *iγ* to account for the broadening of the dispersion due to disorder or correlation effects.

By integrating the phonon out of the action *S*[**Q**_*T*_, **A**_*R*_], one then finds the effective-action *S*_eff_[**A**_*R*_] for the external e.m. field, that is of fourth order in **A**_*R*_. We consider the case in which the polariton at frequency Ω = *ω*_1_ + *ω*_2_ and momentum **k** = **k**_1_ + **k**_2_ is first excited by optical rectification of two incoming optical photons **A**_*R*_(*ω*_1_, **k**_1_) and **A**_*R*_(*ω*_2_, **k**_2_), with k_*i*_ = *n*(*ω*_*i*_)*ω*_*i*_/*c* and *n*(*ω*) the refractive index, and subsequently scatters with a third photon **A**_*R*_(*ω*_3_, **k**_3_), see Fig. 1c. The functional derivative of the effective action with respect to the scattered optical field **A**_*R*_(*ω*_4_, **k**_4_) gives the outgoing nonlinear current, i.e. **J**^(3)^(*ω*_4_, **k**_4_) = ∫ **A**_*R*_(*ω*_1_, **k**_1_)**A**_*R*_(*ω*_2_, **k**_2_)K^(3)^(*ω*_1_ + *ω*_2_, **k**_1_ + **k**_2_)**A**_*R*_(*ω*_3_, **k**_3_)*δ*(*ω*_1_ + *ω*_2_ + *ω*_3_ − *ω*_4_)*dω*_1_*dω*_2_*dω*_3_, where

2 $${{\rm{K}}}^{(3)}(\Omega ,{\bf{k}})\propto {{\mathcal{R}}}^{2}{D}_{{\rm{Q}}}(\Omega ,{\bf{k}})$$

is the third-order nonlinear kernel. Notice that since K^(3)^ is proportional to the PhP propagator Eq. (1), the current is non-negligible only when the hybrid mode has sizeable phonon character at frequency Ω = *ω*_1_ + *ω*_2_.

### Three-wave mixing interaction

In the TWM case the coherent excitation of the IR-active phonon is accomplished by an external THz field **A**_*p*_ resonant with the lattice mode. In this case the THz field cannot be integrated out of the action, since one is interested in a nonlinear current **J**^(2)^ that scales linearly with **A**_*p*_. By taking into account a Raman-like detection with a visible probe field **A**_*R*_, the full TWM light-phonon action reads *S*[**Q**_*T*_, **A**_*R*_, **A**_*p*_] = *S*_0_[**Q**_*T*_] + *S*_*R*_[**Q**_*T*_, **A**_*R*_] + *S*_IR_[**Q**_*T*_, **A**_*p*_]. By integrating out the transverse bare phonon one finds the effective-action *S*_eff_[**A**_*R*_, **A**_*p*_] for the e.m. fields, from which we retain only terms at first order in the THz pump **A**_*p*_(*ω*_1_, **k**_1_) and second order in the optical field **A**_*R*_(*ω*_2_, **k**_2_)**A**_*R*_(*ω*_3_, **k**_3_), see Fig. 1d. The nonlinear current reads **J**^(2)^(*ω*_3_, **k**_3_) = ∫ **A**_*p*_(*ω*_1_, **k**_1_)K^(2)^(*ω*_1_)**A**_*R*_(*ω*_2_, **k**_2_)*δ*(*ω*_1_ + *ω*_2_ − *ω*_3_)*dω*_1_*dω*_2_, where the second-order nonlinear kernel

3 $${{\rm{K}}}^{(2)}(\Omega )\propto \Omega ({\rm{Z}}{\mathcal{R}}){D}_{0}(\Omega )$$

scales as a rank-3 tensor, coming from the interplay between the tensors Z and ${\mathcal{R}}$, mediated by the noncentrosymmetric bare phonon mode. The tensorial structure of the kernel is closely related to the symmetry properties of the sample^17,20,33^, and it determines the selection rules for the corresponding nonlinear response. Indeed, as we detail in Supplementary Note 2, the signal can be strongly suppressed, and even disappear, in specific polarization geometries according to the structure of the crystal^30^. In contrast to the FWM case, the nonlinear kernel in Eq. (3) is proportional to the *bare* phonon propagator. Nonetheless, as we shall discuss below, the polariton affects the response via the propagation effects of the THz field **A**_*p*_, that directly controls the nonlinear current **J**^(2)^.

### Propagation effects

The nth-order nonlinear current **J**^(n)^(*ω*, *z*) acts as a source term in Maxwell’s equation for the gauge field, ${\partial }_{z}^{2}{\bf{A}}(\omega ,z)+{n}^{2}(\omega ){\omega }^{2}{\bf{A}}(\omega ,z)/{c}^{2}=-4\pi {{\bf{J}}}^{({\rm{n}})}(\omega ,z)/c$. By treating the nonlinear current as a perturbation, we derive the leading-order perturbative solution **A**^[1]^(*ω*, *z*) in a confined region 0 < *z* < *d*, where *d* is the sample thickness. The procedure is detailed in Supplementary Note 3. The solution explicitly accounts for a possible finite momentum mismatch Δk between the interacting e.m. fields. When computing e.g. the transmitted field **A**^[1]^(*ω*, *d*^+^) in the TWM case, the phase-matching condition Δk = 0 requires *n*(*ω*_1_)*ω*_1_/*c* = *n*(*ω*)*ω*/*c* − *n*(*ω*_2_)*ω*_2_/*c*. Here *ω*_1_ denotes the frequency of the incoming THz field while *ω*_2_ and *ω* denote the frequencies of the incoming and outgoing visible light pulses respectively, with *ω* = *ω*_1_ + *ω*_2_. Considering an approximately constant optical refractive index *n*(*ω*) ≃ *n*(*ω*_2_) ≡ *n*_eV_, the phase-matching condition is *n*(*ω*_1_)*ω*_1_/*c* = *n*_eV_*ω*_1_/*c*, which admits a simple graphical representation in the (*ω*_1_, k_1_)-plane as the intersection between the PhP dispersion and the curve *ω*_1_ = *c*k_1_/*n*_eV_^19^, which typically occurs at momenta in which the PhP is of hybrid character. If one is interested in the reflected field **A**^[1]^(*ω*, 0^−^), instead, one should replace *ω*_2_ → − *ω*_2_ in the phase-matching condition and the two curves intersect at high momenta where the hybrid PhP character is lost.

### Pump-probe experiments

In pump-probe protocols variations in the probe field are collected as function of the pump-probe time delay *t*_*pp*_, at fixed observation time. As detailed in Methods, the Fourier transform of the measured signal in transmission ${{\bf{A}}}_{{\rm{tr}}}({\omega }_{pp})$ or reflection **A**_ref_(*ω*_*pp*_) geometry can be directly related to **A**^[1]^(*ω*, *d*^+^) and **A**^[1]^(*ω*, 0^−^) respectively.

**Fig. 2:**
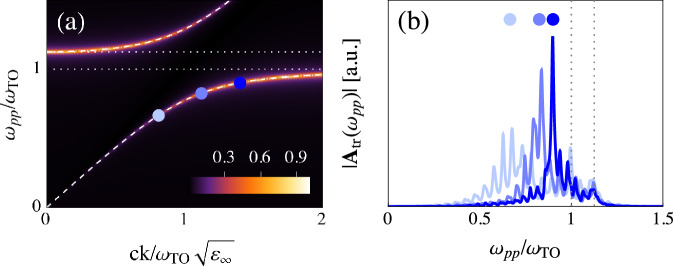
Optical pump-optical probe experiments in transmission geometry with collinear pulses. **a** Phase-matched points for different values of ${n}_{{\rm{eV}}}/\sqrt{{\varepsilon }_{\infty }}$, changed between 1.20 (light blue), 1.35 (blue) and 1.54 (dark blue). **b** Corresponding transmitted intensity $| {{\bf{A}}}_{{\rm{tr}}}({\omega }_{pp})|$. In both panels, *ω*_LO_ = 1.125*ω*_TO_, *γ* = 0.01*ω*_TO_ and $d=50c/{\omega }_{{\rm{TO}}}\sqrt{{\varepsilon }_{\infty }}$ (order of 100 μm in typical samples). Dotted lines highlight *ω*_TO_ and *ω*_LO_.

We first consider optical pump-optical probe FWM experiments (ISRS), see Supplementary Note 5. In this case, the nonlinear current is a convolution of the kernel K^(3)^ in Eq. (2) with the optical e.m. field **A**_*R*_ inside the sample. At visible frequencies *ω* ≫ *ω*_TO_ the PhP does not quantitatively affect the refractive index *n*(*ω*), whose dispersion is essentially controlled by electronic processes. Because of this, the resulting nonlinear signal can be understood as a direct measurement of the phonon character of the PhP encoded in the dressed phonon propagator Eq. (1), with momentum dictated by the phase-matching condition Δk = 0 that allows one to reconstruct the dispersion^3,9–18^. Figure 2 shows a qualitative result for $| {{\bf{A}}}_{{\rm{tr}}}({\omega }_{pp})|$ in optical pump-probe experiments. The phase-matched frequency is tuned by changing the central frequency of the pulses, and thus *n*_eV_. Notice that the intensity of the main peak grows approaching *ω*_TO_, as the phonon character of the PhP progressively increases. The satellite peaks are associated with screening effects at the interface and Fabry-Perot internal reflections.

**Fig. 3:**
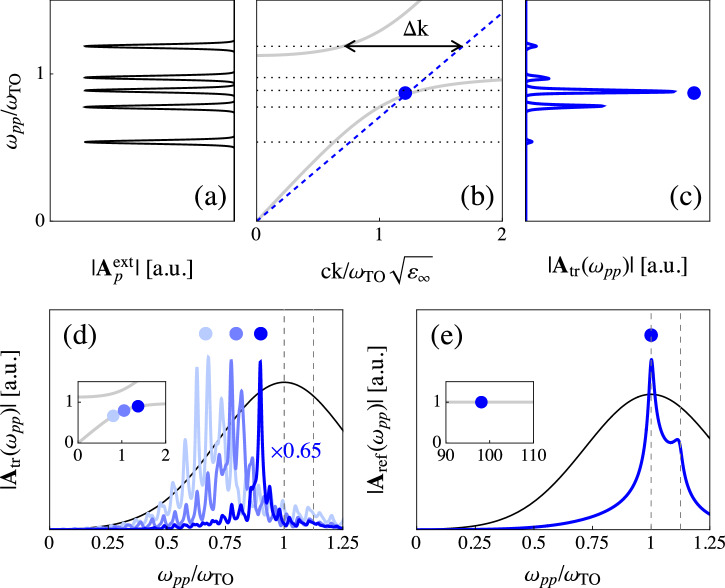
THz pump-optical probe experiments with collinear pulses. **a** Spectral content of narrowband pump pulses with different central frequencies *ω*_*p*_. **b** Phase-matched point in transmission geometry identified by the intersection between the PhP dispersion (gray) and *c*k/*n*_eV_ (blue dashed). Dotted horizontal lines are guides to the eye at *ω*_*p*_. Black arrow represents the momentum mismatch Δk for one of the pumps. **c** Transmitted signal $| {{\bf{A}}}_{{\rm{tr}}}({\omega }_{pp})|$ for the different pump pulses shown in panel (**a**). The blue dot marks the phase-matched frequency. **d** Transmitted signal $| {{\bf{A}}}_{{\rm{tr}}}({\omega }_{pp})|$ induced by a broadband pump pulse (black). ${n}_{{\rm{eV}}}/\sqrt{{\varepsilon }_{\infty }}$ is changed between 1.20 (light blue), 1.32 (blue) and 1.54 (dark blue) to move the phase-matched frequency (dots) along the dispersion (inset). Vertical dashed lines highlight *ω*_TO_ and *ω*_LO_. **e** Reflected signal ∣**A**_ref_(*ω*_*pp*_)∣ induced by a broadband pump pulse (black). The phase-matched frequency (dot) is fixed by ${n}_{{\rm{eV}}}/\sqrt{{\varepsilon }_{\infty }}=1.41$ and falls on the high-momentum region of the dispersion (inset). Vertical dashed lines highlight *ω*_TO_ and *ω*_LO_. In all panels, *ω*_LO_ = 1.125*ω*_TO_ and *γ* = 0.01*ω*_TO_. Sample thickness is $d=50c/{\omega }_{{\rm{TO}}}\sqrt{{\varepsilon }_{\infty }}$ for transmission and *d* → *∞* for reflection geometries.

In THz pump-optical probe TWM experiments the presence of the PhP makes the refractive index for the THz pump pulse **A**_*p*_ highly dispersive. Because of this, even though the polariton does not explicitly appear in the K^(2)^ kernel Eq. (3), it still modulates the propagation of the THz field inside the material and, consequently, of the generated nonlinear signal. We model the incident THz pump field as a Gaussian spectrum centered at *ω*_*p*_ with pulse duration *τ*. The polarization of the pump and probe pulses are fixed to maximize the signal according to the symmetry properties of the kernel Eq. (3), while the probe frequency is tuned to adjust the phase-matching condition Δk = 0 and move along the dispersion branches. As discussed above, to access the PhP dispersion in the region where the light-phonon mixing is strong, experiments in transmission geometry are preferable as the phase-matching condition is usually realized at small momenta ${\rm{k}} \sim \sqrt{{\varepsilon }_{\infty }}{\omega }_{{\rm{TO}}}/c$. In this case the signal in frequency space reads

4 $$\begin{array}{lll}{{\bf{A}}}_{{\rm{tr}}}({\omega }_{pp})\,=\,\mathop{\sum }\limits_{{\sigma }_{1},{\sigma }_{2}}^{\pm 1}{{\bf{A}}}_{{\sigma }_{1}}({\omega }_{pp}){{\rm{K}}}^{(2)}({\omega }_{pp})\int\,d\omega {{\bf{A}}}_{{\sigma }_{2}}(\omega -{\omega }_{pp})\\\qquad\qquad \times\, \frac{2\pi f(\omega )t(\omega )}{\omega }{e}^{i\frac{(n(\omega )+1)\omega d}{c}}\left[\mathop{\sum }\limits_{\alpha }^{\pm 1}{r}_{\alpha }(\omega )\frac{1-{e}^{i\Delta {{\rm{k}}}_{\alpha }d}}{\Delta {{\rm{k}}}_{\alpha }}\right],\end{array}$$

where the coefficients *t*(*ω*) = 2/(*n*(*ω*) + 1), *r*_±_(*ω*) = (*n*(*ω*) ± 1)/(*n*(*ω*) + 1) and the Fabry-Perot factor $f(\omega )=1/(1-{r}_{-}^{2}(\omega ){e}^{2in(\omega )\omega d/c})$ account for the propagation of the e.m. fields at the interfaces of the sample, **A**_+1_(*ω*) = **A**_*t*_(*ω*) = **A**^ext^(*ω*)*t*(*ω*)*f*(*ω*) and **A**_−1_(*ω*) = **A**_*r*_(*ω*) = **A**^ext^(*ω*)*t*(*ω*)*r*_−_(*ω*)*f*(*ω*)*e*^2*in*(*ω*)*ωd*/*c*^, with ${{\bf{A}}}^{{\rm{ext}}}(\omega )={{\bf{A}}}_{p}^{{\rm{ext}}}(\omega )+{{\bf{A}}}_{R}^{{\rm{ext}}}(\omega )$ the external perturbation. The term in the square bracket is the phase-matching factor, where the momentum mismatch is Δk_*α*_ = ∑_*i*_*σ*_*i*_k_*i*_ − *αn*(*ω*)*ω*/*c*, with k_1_ = *n*(*ω*_*pp*_)*ω*_*pp*_/*c* and k_2_ = *n*(*ω* − *ω*_*pp*_)(*ω* − *ω*_*pp*_)/*c*. In Fig. 3a–c, we show the signal intensity $| {{\bf{A}}}_{{\rm{tr}}}({\omega }_{pp})|$ induced by narrowband THz pump pulses (*ω*_*p*_*τ* ≫ 1) with different central frequencies *ω*_*p*_, while *n*_eV_ is kept fixed. As expected, the response is maximized when the phase-matching condition is fulfilled. Nonetheless, a sizable response is also observed for Δk ≠ 0, underlining the main difference between the coherent nonlinear excitation of PhPs and spontaneous Raman scattering, in which only phase-matched polaritons can be observed^19–21^. We also point out that THz pump-optical probe measurements in the presence of a monochromatic (*ω*_*p*_*τ* → + *∞*) THz pump can be mapped into Raman measurements stimulated by coherent IR radiation^31–36^. In particular, our results are in good agreement with ref. ^31^, where a sizable response is observed even when the frequency *ω*_*p*_ explores the upper polariton branch.

When broadband THz pump pulses (*ω*_*p*_*τ* ≪ 1) are applied, one can in principle access the full frequency range in which the polariton has mixed light-matter character within a single measurement. Figure 3d shows $| {{\bf{A}}}_{{\rm{tr}}}({\omega }_{pp})|$ for a fixed pump field and different optical refractive indices. Analogously to the ISRS case, the main frequency of the oscillations is the phase-matched one, highlighted by dots and redshifted with respect to *ω*_TO_. Nonetheless, in this case the relative height of the peaks cannot be directly linked to the phonon character of the PhP, but rather it scales as the field strength of the pump. In other words, most of the signal follows the spectral content of the THz pulse, with a strong enhancement at the phase-matched frequency. The same conclusions can be drawn when performing calculations with more realistic THz pump profiles, as the ones generated by organic emitters^26,28,48,49^, as we discuss in Supplementary Note 4. We also show that in thin samples ($d\lesssim c/{\omega }_{{\rm{TO}}}\sqrt{{\varepsilon }_{\infty }}$) or when the penetration depth of the optical pulses is small ($\delta \lesssim c/{\omega }_{{\rm{TO}}}\sqrt{{\varepsilon }_{\infty }}$) the phase-matching condition loses significance. In reflection geometry, the signal in frequency space reads

5 $$\begin{array}{ll}{{\bf{A}}}_{{\rm{ref}}}({\omega }_{pp})\,=\,-{{\bf{A}}}_{t}({\omega }_{pp}){{\rm{K}}}^{(2)}({\omega }_{pp})\int\,d\omega {{\bf{A}}}_{t}(\omega -{\omega }_{pp})\\ \qquad\qquad\quad\times \frac{2\pi t(\omega )}{\omega }\left[\frac{1}{{{\rm{k}}}_{1}+{{\rm{k}}}_{2}+n(\omega )\omega /c}\right],\end{array}$$

where we considered the *d* → *∞* limit, as appropriate for typical reflection experiments. Figure 3e shows ∣**A**_ref_(*ω*_*pp*_)∣ resulting from a broadband THz pump. In this case the phase-matching condition is met in the region ${\rm{k}}\gg \sqrt{{\varepsilon }_{\infty }}{\omega }_{{\rm{TO}}}/c$ in which the dispersion of the PhP becomes flat and approaches the bare-phonon frequency *ω*_TO_. As a consequence, the signal is peaked at *ω*_TO_, while a secondary peak at *ω*_LO_ is given by the screening of the THz pump at the interface, in excellent agreement with experimental data^30^.

## Discussion

In this work we provided a microscopic derivation of the nonlinear signal generated by multiwave mixing processes in noncentrosymmetric cubic crystals. We rely on a microscopic derivation of the nonlinear current generated in the sample, based on a many-body effective-action derivation of the optical kernel, and on a perturbative solution of the nonlinear Maxwell’s equations, needed to account for propagation effects and phase-matching conditions. We have shown that the hybrid nature of the PhP enters the response in different ways depending on the experimental protocols. For all-optical FWM interactions we showed that the nonlinear kernel itself maps out the *phonon* component of the PhP, see Eq. (2), while propagation effects play a minor role. This allows one to directly access the hybrid-mode dispersion by tuning the frequency of the visible probe pulse in ISRS measurements. However, when the phase-matching condition moves towards the low-momenta region the signal loses intensity, due to the suppression of the phonon character of the PhP branch, see Fig. 2. In contrast, for THz pump-optical probe TWM protocols the nonlinear kernel itself is proportional to the bare phonon mode, see Eq. (3), and light-phonon hybridization affects the propagation of the THz pump field, leading again to a pronounced peak at the phase-matched frequency. This has the remarkable advantage that for broadband THz pulses one can push the detection towards the low-momenta region, enlarging the phase space of the PhP dispersion accessible with the same experimental configuration. On a general perspective, the present formalism is flexible to include additional coupling channels between the phonon and other matter degrees of freedom, as e.g. anharmonically coupled PhPs^3,12–14,26–29^, or magnon-phonon polaritons^7,50^, as well as to describe higher order IR-phonon excitations^51,52^. Moreover, although we considered cubic crystals as a case study in this manuscript, our formalism can be generalized to anisotropic structures. In such materials, the way light couples with the phonon differs depending on the crystallographic direction, leading to strong birefringence and thus affecting the way in which the polaritons disperse and in which propagation effects modulate the pump-probe response. A careful treatment of anisotropy in our model could help probing and understanding effects such as the highly-directional propagation and the hyperbolic dispersion of the polaritons^1,25,53,54^ in TWM and FWM schemes. As a related possible application, the approach we employed to describe the dispersion of bulk PhPs through Eq. (1) can also be applied to surface PhPs^1,55–57^, in combination with a systematic treatment of vanishing waves in the many-body framework and of their propagation along the surface. Detection of these modes in a large variety of quantum materials has been realized through both TWM and FWM schemes^54,58,59^ but, while a theoretical approach to the FWM detection of these modes has been recently provided^60^, a comprehensive theory that can describe TWM interaction schemes is still lacking. Finally, since the effective-action description incorporates the phonon and light fields on the same footing, our results can be generalized to infer information on light-matter interaction in optical cavities, given the intriguing possibility to investigate cavity polaritons using pump-probe spectroscopy^61–63^.

## Methods

### Measured quantity in pump-probe experiments

In pump-probe experiments, variations in the transmitted or reflected probe field are measured as function of the time delay *t*_*pp*_ between pump and probe pulses. The transmitted and reflected fields become functions of the time delay, **A**^[1]^(*ω*, *d*^+^) → **A**^[1]^(*ω*, *t*_*pp*_, *d*^+^) and **A**^[1]^(*ω*, 0^−^) → **A**^[1]^(*ω*, *t*_*pp*_, 0^−^) respectively, through the external field that appears in **A**_*t*_(*ω*, *t*_*pp*_) and **A**_*r*_(*ω*, *t*_*pp*_), as

6 $${{\bf{A}}}^{{\rm{ext}}}(\omega )\to {{\bf{A}}}^{{\rm{ext}}}(\omega ,{t}_{pp})={{\bf{A}}}_{R}^{{\rm{ext}}}(\omega )+{{\bf{A}}}_{R/p}^{{\rm{ext}}}(\omega ){e}^{-i\omega {t}_{pp}}$$

in the FWM (*R*) and in the TWM (*p*) case. The observation time is fixed, *t* = *t*_gate_, and we set *t*_gate_ = 0 for simplicity. Fixing the observation time corresponds to an integration over *ω* in the frequency domain, meaning that the measured quantity in pump-probe experiments e.g. in transmission configuration ${{\bf{A}}}_{{\rm{tr}}}({t}_{pp})$ reads

7 $${{\bf{A}}}_{{\rm{tr}}}({t}_{pp})={{\bf{A}}}^{[1]}(t=0,{t}_{pp},{d}^{+})=\int\,{{\bf{A}}}^{[1]}(\omega ,{t}_{pp},{d}^{+})d\omega ,$$

while in reflection configuration the measured field **A**_ref_(*t*_*pp*_) reads

8 $${{\bf{A}}}_{{\rm{ref}}}({t}_{pp})={{\bf{A}}}^{[1]}(t=0,{t}_{pp},{0}^{-})=\int\,{{\bf{A}}}^{[1]}(\omega ,{t}_{pp},{0}^{-})d\omega .$$

The spectral content of the pump-probe response can then be found with a Fourier transform to the frequency *ω*_*pp*_.

## Supplementary information

Supplementary Information

## Data Availability

Data have been produced by plotting the analytical formulas derived in the present manuscript. All data and programs are available from the correspondingauthor upon reasonable request.
