# Supplementary material for: Theory of terahertz pump optical probe spectroscopy of phonon polaritons in noncentrosymmetric systems
Source: NPJ Quantum Mater. 2025 May 1;10(1):46. doi: 10.1038/s41535-025-00761-8 (PMC12045802; doi:10.1038/s41535-025-00761-8)
Supplement: Supplementary file 1 — Supplementary Information [file 41535_2025_761_MOESM1_ESM.pdf]

Theory of terahertz pump optical probe spectroscopy of phonon polaritons in noncentrosymmetric systems - Supplementary Material

Niccolò Sellati,[1,*] Jacopo Fiore,[1] Stefano Paolo Villani,[1] Lara Benfatto,[1,†] and Mattia Udina[1,‡]

[1]*Department of Physics, "Sapienza" University of Rome, P.le A. Moro 5, 00185 Rome, Italy*

SUPPLEMENTARY NOTE 1. CHARACTERIZATION OF PHONON POLARITONS IN THE EFFECTIVE-ACTION FORMALISM

To describe the coupling of optical phonons with the internal electromagnetic field in a cubic system we employ the effective-action formalism. In the following we set $\hbar = k_B = 1$ and the take the reduced mass of the oscillator equal to 1. We start from the hamiltonian for an harmonic oscillator of frequency ω_{TO}, which we write as

$$\hat{H} = \frac{1}{2}\sum_{\mathbf{k}}[\omega_{\text{TO}}^2\hat{\mathbf{Q}}_{\mathbf{k}}\hat{\mathbf{Q}}_{-\mathbf{k}} + \hat{\mathbf{P}}_{\mathbf{k}}\hat{\mathbf{P}}_{-\mathbf{k}}], \tag{S1}$$

where the operator $\hat{\mathbf{Q}}_{\mathbf{k}}$ is the phonon displacement and $\hat{\mathbf{P}}_{\mathbf{k}}$ is the phonon momentum. In the following we neglect the $\mathbf{k}$-dependence of the oscillator frequency ω_{TO}, as the hybrid character of the phonon polariton is relevant in a small range of momenta around the Γ-point of the Brillouin zone, in which the uncoupled optical phonon can be taken as non-dispersive. In the many-body path-integral formalism one can equivalently describe the system with a partition function $\mathcal{Z} = \int \mathcal{D}[\mathbf{Q},\mathbf{P}]e^{-S[\mathbf{Q},\mathbf{P}]}$, with the action of the fields $\mathbf{Q}$ and $\mathbf{P}$ that reads

$$S[\mathbf{Q},\mathbf{P}] = \int_0^\beta d\tau \sum_{\mathbf{k}}\left[\frac{1}{2}\omega_{\text{TO}}^2\mathbf{Q}(\tau,\mathbf{k})\mathbf{Q}(\tau,-\mathbf{k}) + \frac{1}{2}\mathbf{P}(\tau,\mathbf{k})\mathbf{P}(\tau,-\mathbf{k}) - i\mathbf{P}(\tau,\mathbf{k})\cdot\partial_\tau\mathbf{Q}(\tau,-\mathbf{k})\right], \tag{S2}$$

where τ is the imaginary time in Matsubara formalism and β is the inverse temperature. We underline that both $\mathbf{Q}$ and $\mathbf{P}$ are vectors in the basis of the three modes, one longitudinal and two transverse optical phonons with respect to the momentum $\mathbf{k}$. Their components, degenerate in a cubic system, can be connected to the displacement and momentum for the Ith atom $\mathbf{u}_I$ and $\mathbf{p}_I$, vectors in the Cartesian basis, through $\text{Q}_s = \sum_I \sqrt{\text{M}_I}\,\mathbf{e}_{s,I}\cdot\mathbf{u}_I$ and $\text{P}_s = \sum_I \mathbf{e}_{s,I}\cdot\mathbf{p}_I/\sqrt{\text{M}_I}$, where M_I is the mass of the Ith atom and $\mathbf{e}_{s,I}$ is the normalized eigenvector of the dynamical matrix corresponding to the sth mode, with degenerate eigenvalue ω_{TO}. Working in the frequency space with $\Omega_m = 2\pi m/\beta$ the bosonic Matsubara frequencies, Eq. (S2) becomes

$$S[\mathbf{Q},\mathbf{P}] = \sum_{i\Omega_m,\mathbf{k}}\left[\frac{1}{2}\omega_{\text{TO}}^2\left|\mathbf{Q}(i\Omega_m,\mathbf{k})\right|^2 + \frac{1}{2}\left|\mathbf{P}(i\Omega_m,\mathbf{k})\right|^2 + \Omega_m\mathbf{P}(i\Omega_m,\mathbf{k})\cdot\mathbf{Q}(-i\Omega_m,-\mathbf{k})\right]. \tag{S3}$$

In the effective-action formalism the propagator of a field can be found as the inverse coefficient of the Gaussian action for the field. To show that Eq. (S3) correctly describes optical phonons, we integrate $\mathbf{P}$ out of the path integral, resulting in a Gaussian action for the field $\mathbf{Q}$:

$$S_0[\mathbf{Q}] = \frac{1}{2}\sum_{i\Omega_m,\mathbf{k}}(\omega_{\text{TO}}^2 + \Omega_m^2)\left|\mathbf{Q}(i\Omega_m,\mathbf{k})\right|^2, \tag{S4}$$

in which one can recognize the well-known bare-phonon propagator once the analytic continuation $i\Omega_m \to \omega + i\gamma$ has been performed, with γ the effective phonon broadening:

$$D_0(\omega) = \frac{2}{\omega_{\text{TO}}^2 - (\omega + i\gamma)^2}. \tag{S5}$$

Notice that up to this point we have not made any distinction between the longitudinal or transverse components of the phonon field, which means that, without any electromagnetic interaction between the ions, they are degenerate at frequency ω_{TO}. In Eq. (S3) one can introduce the linear coupling to the electromagnetic gauge field $\mathbf{A}$ in an effective way, performing the minimal coupling substitution in the Hamiltonian term of the action as

$$\mathbf{P}(i\Omega_m,\mathbf{k}) \to \mathbf{P}(i\Omega_m,\mathbf{k}) - \frac{\text{Z}}{c}\mathbf{A}(i\Omega_m,\mathbf{k}), \tag{S6}$$

where c is the light velocity and Z is a rank-2 tensor, whose matrix elements can be connected to the Born effective charge tensor for the Ith atom, $\tilde{\mathrm{Z}}_I$, through $\mathrm{Z}_{s\lambda} = \sum_I \sum_\tau \mathrm{e}_{s\tau,I}\, \tilde{\mathrm{Z}}_{\tau\lambda,I}/\sqrt{\mathrm{M}_I}$, where λ and τ run over Cartesian components, s is the index of the mode and $\mathrm{e}_{s\tau,I}$ is the τth Cartesian component of $\mathbf{e}_{s,I}$. In cubic systems, Z is a scalar matrix. In introducing the e.m. field we have chosen the Weyl gauge in which the scalar potential is zero. We also add the free electromagnetic field action [1], that reads in Matsubara space

$$\begin{aligned} S_{\text{e.m.}}[\mathbf{A}] =& \frac{1}{8\pi c^2} \sum_{i\Omega_m,\mathbf{k}} \left[\Omega_m^2 \varepsilon_\infty |\mathbf{A}(i\Omega_m,\mathbf{k})|^2 + c^2 |\mathbf{k}\times\mathbf{A}(i\Omega_m,\mathbf{k})|^2\right] \\ =& \frac{1}{c^2} \sum_{i\Omega_m,\mathbf{k}} \left[\Theta^{-1}(i\Omega_m,\mathbf{k})|\mathbf{A}_T(i\Omega_m,\mathbf{k})|^2 + \Lambda^{-1}(i\Omega_m)|\mathrm{A}_L(i\Omega_m,\mathbf{k})|^2\right], \end{aligned} \tag{S7}$$

where ε_∞ is the high-frequency dielectric constant. In the last equivalence we have made explicit that the transverse ($\mathbf{A}_T$) and longitudinal (A_L) components of the field have different bare propagators, $\Theta(i\Omega_m,\mathbf{k}) = 8\pi/(\varepsilon_\infty\Omega_m^2 + c^2|\mathbf{k}|^2)$ and $\Lambda(i\Omega_m,\mathbf{k}) = 8\pi/(\varepsilon_\infty\Omega_m^2)$ respectively. All in all, the action of the system reads

$$S[\mathbf{Q},\mathbf{P},\mathbf{A}] = S[\mathbf{Q},\mathbf{P}] + S_{\text{e.m.}}[\mathbf{A}] + \sum_{i\Omega_m,\mathbf{k}} \left[\frac{\varepsilon_\infty}{8\pi c^2}\Omega_P^2 |\mathbf{A}(i\Omega_m,\mathbf{k})|^2 - \mathbf{P}(i\Omega_m,\mathbf{k})\cdot\frac{\mathrm{Z}}{c}\mathbf{A}(-i\Omega_m,-\mathbf{k})\right], \tag{S8}$$

where Ω_P^2 is the ionic plasma frequency, i.e. the matrix element of the scalar tensor $4\pi\mathrm{Z}^2/\varepsilon_\infty$. We stress that while Eq. (S8) is correct for cubic systems, it can be easily generalized to anisotropic crystals, i.e. nontrivial tensorial structures of Z, with a careful treating of the term $(\mathrm{Z}\mathbf{A}/c)\cdot(\mathrm{Z}\mathbf{A}/c)$ resulting from the minimal coupling Eq. (S6). We then perform the integration of the field $\mathbf{P}$ that gives the action $S[\mathbf{Q},\mathbf{A}] = S_0[\mathbf{Q}] + S_{\text{e.m.}}[\mathbf{A}] + S_{\text{IR}}[\mathbf{Q},\mathbf{A}]$, where

$$S_{\text{IR}}[\mathbf{Q},\mathbf{A}] = \sum_{i\Omega_m,\mathbf{k}} \frac{\Omega_m}{c}\left(\mathrm{Z}\mathbf{A}(i\Omega_m,\mathbf{k})\right)\cdot\mathbf{Q}(-i\Omega_m,-\mathbf{k}). \tag{S9}$$

The structure of the total action is transparent: the first term $S_0[\mathbf{Q}]$ as in Eq. (S4) describes the bare phonon, the second term $S_{\text{e.m.}}[\mathbf{A}]$ as in Eq. (S7) describes the bare e.m. field, whereas the third term $S_{\text{IR}}[\mathbf{Q},\mathbf{A}]$ as in Eq. (S9) describes a linear coupling between the two. Notice that in the latter the transverse (longitudinal) components of $\mathbf{Q}$ only couples to the transverse (longitudinal) components of $\mathbf{A}$, due to the tensor Z being a scalar matrix. When Z has a more complex structure, as is the case of anisotropic materials, such decoupling is not realized, and the problem should be treated more carefully.
This framework offers the possibility to study the phonon polariton in its light character or in its phonon character, provided that one of the two fields is integrated out.

Phonon polariton in the light channel

By integrating out the phonon field $\mathbf{Q}$ from $S[\mathbf{Q},\mathbf{A}]$, one is left with an action of the dressed e.m. field,

$$S'_{\text{e.m.}}[\mathbf{A}] = \frac{1}{8\pi c^2}\sum_{i\Omega_m,\mathbf{k}} \Omega_m^2\varepsilon(i\Omega_m)|\mathrm{A}_L(i\Omega_m,\mathbf{k})|^2 + \left[\Omega_m^2\varepsilon(i\Omega_m) + c^2|\mathbf{k}|^2\right]|\mathbf{A}_T(i\Omega_m,\mathbf{k})|^2, \tag{S10}$$

where the dielectric function is defined as

$$\varepsilon(i\Omega_m) = \varepsilon_\infty\left(1 + \frac{\Omega_P^2}{\omega_{\text{TO}}^2 + \Omega_m^2}\right). \tag{S11}$$

By performing the analytic continuation $i\Omega_m \to \omega + i\gamma$ and by defining the longitudinal-optical phonon frequency $\omega_{\text{LO}}^2 = \omega_{\text{TO}}^2 + \Omega_P^2$, Eq. (S11) can be rewritten as $\varepsilon(\omega) = \varepsilon_\infty((\omega+i\gamma)^2 - \omega_{\text{LO}}^2)/((\omega+i\gamma)^2 - \omega_{\text{TO}}^2)$. This dielectric function is commonly used in the Maxwell's equations to describe phonon polaritons in their light character [2]. Moreover, one finds from this expression the Lyddane-Sachs-Teller relation $\varepsilon_0/\varepsilon_\infty = \omega_{\text{LO}}^2/\omega_{\text{TO}}^2$, with $\varepsilon_0 = \varepsilon(0)$, which encodes the breaking of the degeneracy between the longitudinal-optical and transverse-optical phonons - the LO-TO splitting.
The phonon polariton is described by the transverse component in Eq. (S10). Indeed, one finds the dressed transverse propagator to be

$$\Theta'(i\Omega_m,\mathbf{k}) = \frac{8\pi}{\varepsilon(i\Omega_m)\Omega_m^2 + c^2|\mathbf{k}|^2}, \tag{S12}$$

and its poles after the analytical continuation give the two phonon polariton dispersion branches:

$$\omega_{\pm}^2(\mathbf{k}) = \frac{1}{2}(\omega_{\rm LO}^2 + c^2|\mathbf{k}|^2/\varepsilon_\infty) \pm \frac{1}{2}\sqrt{(\omega_{\rm LO}^2 + c^2|\mathbf{k}|^2/\varepsilon_\infty)^2 - 4\omega_{\rm TO}^2 c^2|\mathbf{k}|^2/\varepsilon_\infty}. \tag{S13}$$

Phonon polariton in the phonon channel

By integrating out the e.m. field $\mathbf{A}$ from $S[\mathbf{Q},\mathbf{A}]$ one is left with the Gaussian action of the dressed phonon field,

$$S_G[\mathbf{Q}] = \frac{1}{2}\sum_{i\Omega_m,\mathbf{k}} (\Omega_m^2 + \omega_{\rm LO}^2)|{\rm Q}_L(i\Omega_m,\mathbf{k})|^2 + \left[\omega_{\rm TO}^2 + \Omega_m^2 + \frac{\Omega_P^2\Omega_m^2}{\Omega_m^2 + \frac{c^2}{\varepsilon_\infty}|\mathbf{k}|^2}\right]|\mathbf{Q}_T(i\Omega_m,\mathbf{k})|^2, \tag{S14}$$

The LO-TO splitting is transparent from the structure of this action, as the longitudinal ${\rm Q}_L$ and the transverse $\mathbf{Q}_T$ components of the phonon field have different propagators and thus different energies. In particular, in the transverse channel the propagator reads

$$D_{\rm Q}(i\Omega_m,\mathbf{k}) = \frac{2}{\omega_{\rm TO}^2 + \Omega_m^2 + \frac{\Omega_m^2\Omega_P^2}{\Omega_m^2 + \frac{c^2}{\varepsilon_\infty}|\mathbf{k}|^2}} = D_0(i\Omega_m)\frac{\varepsilon_\infty\Omega_m^2 + c^2|\mathbf{k}|^2}{\varepsilon(i\Omega_m)\Omega_m^2 + c^2|\mathbf{k}|^2}, \tag{S15}$$

with bare-phonon propagator $D_0(i\Omega_m)$ defined as in Eq. (S5) and dielectric function $\varepsilon(i\Omega_m)$ defined as in Eq. (S11). After performing the analytical continuation, one recovers the two polaritonic dispersion branches Eq. (S13) as poles of this propagator, weighted by the factor $\varepsilon_\infty\omega^2 - c^2|\mathbf{k}|^2$ that appears in the numerator. Eq. (S15) thus offers another perspective on the hybrid mode, as it describes the polariton in its phonon channel. Moreover, one can easily check from the first expression of Eq. (S15) that for $|\mathbf{k}| \gg \sqrt{\varepsilon_\infty}\Omega_m/c$, the corrections given by the e.m. field vanish, and one recovers $D_{\rm Q}(i\Omega_m,\mathbf{k}) \simeq D_0(i\Omega_m)$. One thus understands that the hybrid nature of the polariton is only relevant for small momenta, in the order of $|\mathbf{k}| \sim \sqrt{\varepsilon_\infty}\omega_{\rm TO}/c$. For the longitudinal phonon one finds from Eq. (S14) a momentum-independent propagator, and the mode is at fixed frequency $\omega_{\rm LO}$.
It is worth mentioning that the integration procedure in the effective-action formalism described in this section is completely equivalent to solving the Dyson equation, as diagrammatically depicted e.g. in Fig. 1(b) of the main text. Nonetheless, the formalism used in this manuscript also allows one to study the interaction with external fields in a straightforward way.

SUPPLEMENTARY NOTE 2. PHONON-MEDIATED LIGHT MIXING PROCESSES

In this section we derive, within the path-integral approach developed above, the nonlinear response for four-wave mixing (FWM) and three-wave mixing (TWM) processes mediated by the transverse phonon. The general strategy is to start from the total action $S[\mathbf{Q}_T,\mathbf{A}] = S_0[\mathbf{Q}_T] + S_{\rm e.m.}[\mathbf{A}] + S_{\rm IR}[\mathbf{Q}_T,\mathbf{A}]$ and introduce a nonlinear Raman-like coupling to the gauge field that reads

$$S_R[\mathbf{Q}_T,\mathbf{A}] = \sum_{i\Omega_m,\mathbf{k}}\sum_{i\Omega_n,\mathbf{k}'}\sum_{i\Omega_l,\mathbf{k}''} \mathbf{Q}_T(-i\Omega_m,-\mathbf{k})\mathcal{R}\mathbf{A}(i\Omega_n,\mathbf{k}')\mathbf{A}(i\Omega_l,\mathbf{k}'')\delta_{i\Omega_l,-i\Omega_m-i\Omega_n}\delta_{\mathbf{k}'',-\mathbf{k}-\mathbf{k}'}. \tag{S16}$$

The Kronecker deltas enforce the conservation of energy and momentum in the interaction process. This interaction is mediated by the rank-3 tensor $\mathcal{R}$, which is connected to the phonon Raman tensor for the Ith atom $\tilde{\mathcal{R}}_I$ as $\mathcal{R}_{s\mu\nu} = \sum_I\sum_\tau {\rm e}_{s\tau,I}\tilde{\mathcal{R}}_{\tau\mu\nu,I}/\sqrt{{\rm M}_I}$, analogously to the tensor Z. One should then integrate out of the total action any fluctuating components that are not linked to external e.m. fields, to find the nth order nonlinear current associated with the light-mixing process.

Four-wave mixing

FWM interactions are realized with optical fields $\mathbf{A}_R$ that are Raman-coupled to the phonon. Experimentally, the spectral components of these fields are typically in the eV range, and as such the THz field, linearly coupled to the phonon, can be distinguished from the optical field and thus it can be integrated out from the total action

when computing the response to $\mathbf{A}_R$. Altogether, the action of the system is the sum of $S_G[\mathbf{Q}_T]$ in Eq. (S14) and $S_R[\mathbf{Q}_T, \mathbf{A}_R]$ in Eq. (S16), which reads explicitly

$$S[\mathbf{Q}_T, \mathbf{A}_R] = \sum_{i\Omega_m, \mathbf{k}} D_{\mathrm{Q}}^{-1}(i\Omega_m, \mathbf{k})|\mathbf{Q}_T(i\Omega_m, \mathbf{k})|^2 + \sum_{i\Omega_m, \mathbf{k}} \sum_{i\Omega_n, \mathbf{k}'} \sum_{i\Omega_l, \mathbf{k}''} \mathbf{Q}_T(-i\Omega_m, -\mathbf{k}) \mathcal{R} \mathbf{A}_R(i\Omega_n, \mathbf{k}') \mathbf{A}_R(i\Omega_l, \mathbf{k}'') \delta_{i\Omega_m, -i\Omega_n - i\Omega_l} \delta_{\mathbf{k}, -\mathbf{k}'-\mathbf{k}''}. \tag{S17}$$

The third-order nonlinear current $\mathbf{J}^{(3)}$ can be computed as a functional derivative with respect to the scattered optical pulse of the effective action $S_{\mathrm{eff}}[\mathbf{A}_R]$, obtained after the integration of the matter degrees of freedom $\mathbf{Q}_T$. By performing the integration in Eq. (S17), one is left with the effective action for the external field,

$$S_{\mathrm{eff}}[\mathbf{A}_R] = -\frac{1}{4} \sum_{i\Omega_1, \mathbf{k}_1} \sum_{i\Omega_2, \mathbf{k}_2} \sum_{i\Omega_3, \mathbf{k}_3} \mathbf{A}_R(i\Omega_1, \mathbf{k}_1) \mathbf{A}_R(i\Omega_2, \mathbf{k}_2) [\mathcal{R}^2 D_{\mathrm{Q}}(i\Omega_1 + i\Omega_2, \mathbf{k}_1 + \mathbf{k}_2)] \times \mathbf{A}_R(i\Omega_3, \mathbf{k}_3) \mathbf{A}_R(-i\Omega_1 - i\Omega_2 - i\Omega_3, -\mathbf{k}_1 - \mathbf{k}_2 - \mathbf{k}_3). \tag{S18}$$

After shifting to real frequencies, the nonlinear current reads

$$\mathbf{J}^{(3)}(\omega_4, \mathbf{k}_4) = -\frac{\delta S_{\mathrm{eff}}[\mathbf{A}_R]}{\delta \mathbf{A}_R(-\omega_4, -\mathbf{k}_4)/c} = \int d\omega_1 d\omega_2 \mathbf{A}_R(\omega_1, \mathbf{k}_1) \mathbf{A}_R(\omega_2, \mathbf{k}_2) \mathrm{K}^{(3)}(\omega_1 + \omega_2, \mathbf{k}_1 + \mathbf{k}_2) \mathbf{A}_R(\omega_3, \mathbf{k}_3) \tag{S19}$$

where energy conservation fixes $\omega_1 + \omega_2 + \omega_3 - \omega_4 = 0$ and momentum conservation fixes $\mathbf{k}_1 + \mathbf{k}_2 + \mathbf{k}_3 - \mathbf{k}_4 = 0$, with $\mathbf{k}_i = n(\omega_i)\omega_i/c$ and $n(\omega_i)$ the refractive index. The third-order nonlinear kernel of the process reads

$$\mathrm{K}^{(3)}(\Omega, \mathbf{k}) \propto \mathcal{R}^2 D_{\mathrm{Q}}(\Omega, \mathbf{k}). \tag{S20}$$

Notice that the kernel depends on the dressed phonon polariton propagator Eq. (S15), which means that the generated nonlinear current is relevant only when the polariton has sizable phonon character at frequency $\Omega = \omega_1 + \omega_2$.
In principle, in FWM experiments also the light character of the PhP, described by Eq. (S12), affects the measured response [3]. Nonetheless, its coupling with the external optical field is mediated by electronic excitations and its contribution to the response is therefore expected to be small when the energy of the optical pulses is far from electronic resonances [4].

Three-wave mixing

In the case of TWM interactions the pump pulse $\mathbf{A}_p$ is in the THz range and it is linearly coupled to the transverse phonon. Thus, the former cannot be integrated out, as one is interested in computing a response that scales linearly with $\mathbf{A}_p$. The detection is realized through an optical field $\mathbf{A}_R$, Raman-coupled to the phonon. In this case the action of the system is thus given by the sum of $S_0[\mathbf{Q}_T]$ in Eq. (S4), $S_R[\mathbf{Q}_T, \mathbf{A}_R]$ in Eq. (S16) and $S_{\mathrm{IR}}[\mathbf{Q}_T, \mathbf{A}_p]$ in Eq. (S9). This reads explicitly

$$S[\mathbf{Q}_T, \mathbf{A}_R, \mathbf{A}_p] = \sum_{i\Omega_m, \mathbf{k}} D_0^{-1}(i\Omega_m)|\mathbf{Q}_T(i\Omega_m, \mathbf{k})|^2 + \sum_{i\Omega_m, \mathbf{k}} \frac{\Omega_m}{c} \big(\mathrm{Z}\mathbf{A}_p(i\Omega_m, \mathbf{k})\big) \cdot \mathbf{Q}_T(-i\Omega_m, -\mathbf{k}) + \sum_{i\Omega_m, \mathbf{k}} \sum_{i\Omega_n, \mathbf{k}'} \sum_{i\Omega_l, \mathbf{k}''} \mathbf{Q}_T(-i\Omega_m, -\mathbf{k}) \mathcal{R} \mathbf{A}_R(i\Omega_n, \mathbf{k}') \mathbf{A}_R(i\Omega_l, \mathbf{k}'') \delta_{i\Omega_m, -i\Omega_n - i\Omega_l} \delta_{\mathbf{k}, -\mathbf{k}'-\mathbf{k}''}. \tag{S21}$$

By performing the integration of $\mathbf{Q}_T$ in Eq. (S21), one is left with all the terms coming from $(\mathbf{A}_p + \mathbf{A}_R^2)^2$. Since we are interested in describing TWM processes we retain only the term of first order in the THz pump and of second order in the optical field:

$$S_{\mathrm{eff}}[\mathbf{A}_R, \mathbf{A}_p] = -\frac{1}{2c} \sum_{i\Omega_m, \mathbf{k}} \sum_{i\Omega_n, \mathbf{k}'} \Omega_m \mathbf{A}_p(i\Omega_m, \mathbf{k}) \big(\mathrm{Z}\mathcal{R} D_0(i\Omega_m)\big) \mathbf{A}_R(i\Omega_n, \mathbf{k}') \mathbf{A}_R(-i\Omega_m - i\Omega_n, -\mathbf{k} - \mathbf{k}'). \tag{S22}$$

After shifting to real frequencies, the nonlinear current reads

$$\mathbf{J}^{(2)}(\omega_3, \mathbf{k}_3) = -\frac{\delta S_{\mathrm{eff}}[\mathbf{A}_R, \mathbf{A}_p]}{\delta \mathbf{A}_R(-\omega_3, -\mathbf{k}_3)/c} = \int d\omega_1 \mathbf{A}_p(\omega_1, \mathbf{k}_1) \mathrm{K}^{(2)}(\omega_1) \mathbf{A}_R(\omega_2, \mathbf{k}_2), \tag{S23}$$

where the conservation of energy and momentum fix $\omega_1 + \omega_2 - \omega_3 = 0$ and $\mathbf{k}_1 + \mathbf{k}_2 - \mathbf{k}_3 = 0$ respectively. The second-order nonlinear kernel of the process reads

$$\mathrm{K}^{(2)}(\Omega) \propto \Omega(\mathrm{Z}\mathcal{R})D_0(\Omega). \tag{S24}$$

The kernel $\mathrm{K}^{(2)}$ is a rank-3 tensor, resulting from the interplay between the rank-2 tensor Z and the rank-3 tensor $\mathcal{R}$, mediated by the noncentrosymmetric phonon mode, i.e. both IR- and Raman-active. By explicitly writing the indices, the tensorial structure of the kernel is given by

$$\mathrm{K}^{(2)}_{\lambda\mu\nu} \propto \sum_s \mathrm{Z}_{\lambda s}\mathcal{R}_{s\mu\nu}. \tag{S25}$$

Thus, the structure of the response kernel depends uniquely on the symmetry of the sample: e.g., for a zincblende structure $\mathrm{K}^{(2)}_{\lambda\mu\nu} \propto \epsilon_{\lambda\mu\nu}$, where $\epsilon_{\lambda\mu\nu}$ is the Levi-Civita tensor. Within the same tensorial notation, one can recast Eq. (S23) as

$$\mathrm{J}^{(2)}_{\nu}(\omega_3, \mathbf{k}_3) \propto \int d\omega_1 \sum_{\lambda\mu} \mathrm{A}_{p,\lambda}(\omega_1, \mathbf{k}_1)\mathrm{K}^{(2)}_{\lambda\mu\nu}(\omega_1, \mathbf{k}_1)\mathrm{A}_{R,\mu}(\omega_2, \mathbf{k}_2). \tag{S26}$$

This expression makes it explicit that the geometry of the experimental setup, i.e. the interplay between the tensorial structure of the kernel and the polarizations of the e.m. fields, plays a crucial role in the TWM process as it determines the selection rules for the related response. As an example we consider a collinear TWM experiment on a zincblende crystal grown along the crystallographic [100] direction, with external pulses propagating in the same direction, as e.g. in Ref. [5]. If the THz pulse $\mathbf{A}_p$ is polarized along the [011] direction and the incident optical pulse $\mathbf{A}_R$ is polarized along the [001] direction, Eq. (S26) states that no current is generated along the transverse directions [001] and [010], that is, there is no response, in agreement with experimental data of Ref. [5].

SUPPLEMENTARY NOTE 3. PROPAGATION EFFECTS ON THE NONLINEAR SIGNAL IN THREE-WAVE MIXING PROCESSES

Once the nonlinear current is generated inside the material, it acts as a source for the nonlinear electric field that propagates inside and at the boundaries of the material and is ultimately detected outside the sample. As the propagation itself affects the signal, a systematic treatment of it is needed in order to avoid misinterpretations of the experimental spectra. In the following we consider the external e.m. fields to be collinear and at normal incidence on the sample. For simplicity of notation we start from the TWM case, and we generalize the equations to the FWM case at the end of this Section.

Three-wave mixing

We consider a material in the region of space $0 < z < d$, with d the sample thickness, characterized by a finite second-order nonlinear kernel $\mathrm{K}^{(2)}(\omega_1, \omega_2)$. Let us consider a generic e.m. field $\mathbf{A}(\omega, z)$ traveling along z and inducing a nonlinear current

$$\mathbf{J}^{(2)}(\omega, z) = \int d\omega_1 d\omega_2 \mathbf{A}(\omega_1, z)\mathrm{K}^{(2)}(\omega_1, \omega_2)\mathbf{A}(\omega_2, z)\delta(\omega - \omega_1 - \omega_2), \tag{S27}$$

which acts as a source term in Maxwell's equations for the nonlinear signal:

$$\begin{cases} \partial_z^2 \mathbf{A}(\omega, z) + \frac{\omega^2}{c^2}\mathbf{A}(\omega, z) = 0 & (z < 0, z > d), \\ \partial_z^2 \mathbf{A}(\omega, z) + \frac{n^2(\omega)\omega^2}{c^2}\mathbf{A}(\omega, z) = -\frac{4\pi}{c}\mathbf{J}^{(2)}(\omega, z) & (0 < z < d). \end{cases} \tag{S28}$$

In principle the equation within the sample can only be solved self-consistently due to the presence of $\mathbf{A}(\omega, z)$ on the right hand side of the source term Eq. (S27). Nonetheless, since nonlinear effects are usually smaller with respect to linear ones, which are already taken into account in the definition of $n(\omega)$, it is possible to solve the equation with a perturbative approach. We introduce a fictitious small parameter η such that

$$\mathrm{K}^{(2)}(\omega_1, \omega_2) = \eta\tilde{\mathrm{K}}^{(2)}(\omega_1, \omega_2) \tag{S29}$$

and

$$\mathbf{A}(\omega, z) = \sum_{n} \eta^n \mathbf{A}^{[n]}(\omega, z). \tag{S30}$$

Eq. (S27) can then be written as

$$\mathbf{J}^{(2)}(\omega, z) = \sum_{n,m} \eta^{n+m+1} \int d\omega_1 d\omega_2 (\omega_1, \omega_2) \mathbf{A}^{[n]}(\omega_1, z) \tilde{\mathrm{K}}^{(2)}(\omega_1, \omega_2) \mathbf{A}^{[m]}(\omega_2, z) \delta(\omega - \omega_1 - \omega_2). \tag{S31}$$

We can then solve Eq. (S28) for every order of η. At zero-th order the system is

$$\begin{cases} \partial_z^2 \mathbf{A}^{[0]}(\omega, z) + \frac{\omega^2}{c^2} \mathbf{A}^{[0]}(\omega, z) = 0 & (z < 0, z > d), \\ \partial_z^2 \mathbf{A}^{[0]}(\omega, z) + \frac{n^2(\omega)\omega^2}{c^2} \mathbf{A}^{[0]}(\omega, z) = 0 & (0 < z < d), \end{cases} \tag{S32}$$

which describes the linear propagation of the field inside the material. The solution in the region $0 < z < d$ is

$$\mathbf{A}^{[0]}(\omega, z) = \mathbf{A}_t(\omega) e^{in(\omega)\omega z/c} + \mathbf{A}_r(\omega) e^{-in(\omega)\omega z/c}, \tag{S33}$$

where $\mathbf{A}_t(\omega) = \mathbf{A}^{\mathrm{ext}}(\omega) t(\omega) f(\omega)$ and $\mathbf{A}_r(\omega) = \mathbf{A}^{\mathrm{ext}}(\omega) t(\omega) r(\omega) f(\omega) e^{2in(\omega)\omega d/c}$, with transmission coefficient $t(\omega) = 2/(1+n(\omega))$, reflection coefficient $r(\omega) = (n(\omega)-1)/(n(\omega)+1)$ and Fabry-Perot factor $f(\omega) = 1/(1-r^2(\omega)e^{2in(\omega)\omega d/c})$. Here $\mathbf{A}^{\mathrm{ext}}(\omega)$ is the spectrum of the external incident field.
At first order in η, the system reads

$$\begin{cases} \partial_z^2 \mathbf{A}^{[1]}(\omega, z) + \frac{\omega^2}{c^2} \mathbf{A}^{[1]}(\omega, z) = 0 & (z < 0, z > d), \\ \partial_z^2 \mathbf{A}^{[1]}(\omega, z) + \frac{n^2(\omega)\omega^2}{c^2} \mathbf{A}^{[1]}(\omega, z) = -\frac{4\pi}{c} \mathbf{J}^{(2)}_{[1]}(\omega, z) & (0 < z < d), \end{cases} \tag{S34}$$

where

$$\mathbf{J}^{(2)}_{[1]}(\omega, z) = \int d\omega_1 d\omega_2 \mathbf{A}^{[0]}(\omega_1, z) \mathrm{K}^{(2)}(\omega_1, \omega_2) \mathbf{A}^{[0]}(\omega_2, z) \delta(\omega - \omega_1 - \omega_2). \tag{S35}$$

The solution of Eq. (S34) in $0 < z < d$ can be written as

$$\mathbf{A}^{[1]}(\omega, z) = \mathbf{A}^u(\omega, z) + \mathbf{B}(\omega) e^{in(\omega)\omega z/c} + \mathbf{C}(\omega) e^{-in(\omega)\omega z/c} \tag{S36}$$

where $\mathbf{B}(\omega)$ and $\mathbf{C}(\omega)$ are the coefficients of the forward and backward propagating fields respectively, determined by boundary conditions. $\mathbf{A}^u(\omega, z)$ is a unique particular solution to the differential equation, which reads

$$\mathbf{A}^u(\omega, z) = \frac{4\pi}{c} \frac{1}{2\pi} \int d\mathrm{k} \frac{e^{i\mathrm{k}z}}{\mathrm{k}^2 - n^2(\omega)\omega^2/c^2} \mathbf{J}^{(2)}_{[1]}(\omega, \mathrm{k}), \tag{S37}$$

with $\mathbf{J}^{(2)}_{[1]}(\omega, \mathrm{k})$ obtained from Eq. (S35) by insertion of the solution Eq. (S33) as

$$\begin{aligned} \mathbf{J}^{(2)}_{[1]}(\omega, \mathrm{k}) &= \int_0^d dz\, \mathbf{J}^{(2)}_{[1]}(\omega, z) e^{-i\mathrm{k}z} \\ &= \int d\omega_1 d\omega_2 \Big[\mathbf{A}_t(\omega_1) \mathrm{K}^{(2)}(\omega_1, \omega_2) \mathbf{A}_t(\omega_2) \frac{1 - e^{-i(\mathrm{k}-\mathrm{k}_1-\mathrm{k}_2)d}}{i(\mathrm{k}-\mathrm{k}_1-\mathrm{k}_2)} + \mathbf{A}_t(\omega_1) \mathrm{K}^{(2)}(\omega_1, \omega_2) \mathbf{A}_r(\omega_2) \frac{1 - e^{-i(\mathrm{k}-\mathrm{k}_1+\mathrm{k}_2)d}}{i(\mathrm{k}-\mathrm{k}_1+\mathrm{k}_2)} \\ &\qquad \mathbf{A}_r(\omega_1) \mathrm{K}^{(2)}(\omega_1, \omega_2) \mathbf{A}_t(\omega_2) \frac{1 - e^{-i(\mathrm{k}+\mathrm{k}_1-\mathrm{k}_2)d}}{i(\mathrm{k}+\mathrm{k}_1-\mathrm{k}_2)} + \mathbf{A}_r(\omega_1) \mathrm{K}^{(2)}(\omega_1, \omega_2) \mathbf{A}_r(\omega_2) \frac{1 - e^{-i(\mathrm{k}+\mathrm{k}_1+\mathrm{k}_2)d}}{i(\mathrm{k}+\mathrm{k}_1+\mathrm{k}_2)} \Big] \\ &\quad \times \delta(\omega - \omega_1 - \omega_2) \end{aligned} \tag{S38}$$

where we defined $\mathrm{k}_i = \mathrm{k}(\omega_i) = n(\omega_i)\omega_i/c$. The integral over k in Eq. (S37) can be solved with the residue theorem and the unique solution reads explicitly

$$\begin{aligned}
\mathbf{A}^u(\omega, z) = \frac{4\pi}{c} \int d\omega_1 d\omega_2 \; \delta(\omega - \omega_1 - \omega_2) \\
\times \Bigg[\mathbf{A}_t(\omega_1)\mathrm{K}^{(2)}(\omega_1,\omega_2)\mathbf{A}_t(\omega_2) \Bigg(\frac{e^{i(\mathrm{k}_1+\mathrm{k}_2)z}}{(\mathrm{k}_1+\mathrm{k}_2)^2 - \frac{n^2(\omega)\omega^2}{c^2}} + \frac{e^{in(\omega)\omega z/c}}{\frac{2n(\omega)\omega}{c}\left(\frac{n(\omega)\omega}{c} - \mathrm{k}_1 - \mathrm{k}_2\right)} + \frac{e^{i(\mathrm{k}_1+\mathrm{k}_2)d} e^{-in(\omega)\omega(z-d)/c}}{\frac{2n(\omega)\omega}{c}\left(\frac{n(\omega)\omega}{c} + \mathrm{k}_1 + \mathrm{k}_2\right)} \Bigg) \\
+ \mathbf{A}_t(\omega_1)\mathrm{K}^{(2)}(\omega_1,\omega_2)\mathbf{A}_r(\omega_2) \Bigg(\frac{e^{i(\mathrm{k}_1-\mathrm{k}_2)z}}{(\mathrm{k}_1-\mathrm{k}_2)^2 - \frac{n^2(\omega)\omega^2}{c^2}} + \frac{e^{in(\omega)\omega z/c}}{\frac{2n(\omega)\omega}{c}\left(\frac{n(\omega)\omega}{c} - \mathrm{k}_1 + \mathrm{k}_2\right)} + \frac{e^{i(\mathrm{k}_1-\mathrm{k}_2)d} e^{-in(\omega)\omega(z-d)/c}}{\frac{2n(\omega)\omega}{c}\left(\frac{n(\omega)\omega}{c} + \mathrm{k}_1 - \mathrm{k}_2\right)} \Bigg) \\
+ \mathbf{A}_r(\omega_1)\mathrm{K}^{(2)}(\omega_1,\omega_2)\mathbf{A}_t(\omega_2) \Bigg(\frac{e^{i(\mathrm{k}_2-\mathrm{k}_1)z}}{(\mathrm{k}_2-\mathrm{k}_1)^2 - \frac{n^2(\omega)\omega^2}{c^2}} + \frac{e^{in(\omega)\omega z/c}}{\frac{2n(\omega)\omega}{c}\left(\frac{n(\omega)\omega}{c} + \mathrm{k}_1 - \mathrm{k}_2\right)} + \frac{e^{i(\mathrm{k}_2-\mathrm{k}_1)d} e^{-in(\omega)\omega(z-d)/c}}{\frac{2n(\omega)\omega}{c}\left(\frac{n(\omega)\omega}{c} - \mathrm{k}_1 + \mathrm{k}_2\right)} \Bigg) \\
+ \mathbf{A}_r(\omega_1)\mathrm{K}^{(2)}(\omega_1,\omega_2)\mathbf{A}_r(\omega_2) \Bigg(\frac{e^{-i(\mathrm{k}_1+\mathrm{k}_2)z}}{(\mathrm{k}_1+\mathrm{k}_2)^2 - \frac{n^2(\omega)\omega^2}{c^2}} + \frac{e^{in(\omega)\omega z/c}}{\frac{2n(\omega)\omega}{c}\left(\frac{n(\omega)\omega}{c} + \mathrm{k}_1 + \mathrm{k}_2\right)} + \frac{e^{-i(\mathrm{k}_1+\mathrm{k}_2)d} e^{-\mathrm{i}n(\omega)\omega(z-d)/c}}{\frac{2n(\omega)\omega}{c}\left(\frac{n(\omega)\omega}{c} - \mathrm{k}_1 - \mathrm{k}_2\right)} \Bigg) \Bigg],
\end{aligned} \tag{S39}$$

The solution of the differential equation Eq. (S34) outside the sample is $\mathbf{A}^{[1]}(\omega, z > d) = \mathbf{B}'(\omega)e^{i\omega z/c}$ and $\mathbf{A}^{[1]}(\omega, z < 0) = \mathbf{C}'(\omega)e^{-i\omega z/c}$. Applying continuity conditions for the fields and their derivatives at the boundaries,

$$\begin{cases}
\mathbf{C}'(\omega) = \mathbf{A}^u(\omega, 0) + \mathbf{B}(\omega) + \mathbf{C}(\omega) \\
-\frac{i\omega}{c}\mathbf{C}'(\omega) = \mathbf{A}^{u\prime}(\omega, 0) + \frac{in(\omega)\omega}{c}\big(\mathbf{B}(\omega) - \mathbf{C}(\omega)\big) \\
\mathbf{B}'(\omega)e^{i\omega d/c} = \mathbf{A}^u(\omega, d) + \mathbf{B}(\omega)e^{in(\omega)\omega d/c} + \mathbf{C}(\omega)e^{-in(\omega)\omega d/c} \\
\frac{i\omega}{c}\mathbf{B}'(\omega)e^{i\omega d/c} = \mathbf{A}^{u\prime}(\omega, d) + \frac{in(\omega)\omega}{c}\big(\mathbf{B}(\omega)e^{in(\omega)\omega d/c} - \mathbf{C}(\omega)e^{-in(\omega)\omega d/c}\big),
\end{cases} \tag{S40}$$

one can find the coefficients of the transmitted $\mathbf{B}'$ and reflected $\mathbf{C}'$ fields, that read explicitly

$$\begin{aligned}
\mathbf{B}'(\omega) = -\frac{f(\omega)}{(n(\omega)+1)^2}\Big[& 2n(\omega)e^{in(\omega)\omega d/c}\big(\mathbf{A}^u(\omega,0) - \frac{ic}{\omega}\mathbf{A}^{u\prime}(\omega,0)\big) \\
& -(n(\omega)+1)\big(n(\omega)\mathbf{A}^u(\omega,d) - \frac{ic}{\omega}\mathbf{A}^{u\prime}(\omega,d)\big) \\
& +(n(\omega)-1)e^{2in(\omega)\omega d/c}\big(n(\omega)\mathbf{A}^u(\omega,d) + \frac{ic}{\omega}\mathbf{A}^{u\prime}(\omega,d)\big)\Big]
\end{aligned} \tag{S41}$$

and

$$\begin{aligned}
\mathbf{C}'(\omega) = -\frac{f(\omega)}{(n(\omega)+1)^2}\Big[& (n(\omega)-1)e^{2in(\omega)\omega d/c}\big(n(\omega)\mathbf{A}^u(\omega,0) - \frac{ic}{\omega}\mathbf{A}^{u\prime}(\omega,0)\big) \\
& -(n(\omega)+1)\big(n(\omega)\mathbf{A}^u(\omega,0) + \frac{ic}{\omega}\mathbf{A}^{u\prime}(\omega,0)\big) \\
& +2n(\omega)e^{in(\omega)\omega d/c}\big(\mathbf{A}^u(\omega,d) + \frac{ic}{\omega}\mathbf{A}^{u\prime}(\omega,d)\big)\Big].
\end{aligned} \tag{S42}$$

Usually in reflection experiments the sample thickness is much greater than the wavelength of the radiation $d \gg \lambda$ and of the penetration depth of the optical pulses $d \gg \delta$. In this case Eq. (S42) can be approximated as

$$\mathbf{C}'(\omega) \simeq \frac{1}{n(\omega)+1}\Big[n(\omega)\mathbf{A}^u(\omega,0) + \frac{ic}{\omega}\mathbf{A}^{u\prime}(\omega,0)\Big]. \tag{S43}$$

Using Eq. (S39) in Eqs. (S41) and (S43) one finds

$$\begin{aligned}\mathbf{B}'(\omega) =& \frac{4\pi}{\omega}\frac{f(\omega)}{n(\omega)+1}e^{in(\omega)\omega d/c}\int d\omega_1 d\omega_2\ \delta(\omega-\omega_1-\omega_2)\\ &\times\Bigg[\mathbf{A}_t(\omega_1)\mathrm{K}^{(2)}(\omega_1,\omega_2)\mathbf{A}_t(\omega_2)\left(\frac{e^{i(\mathrm{k}_1+\mathrm{k}_2-n(\omega)\omega/c)d}-1}{\mathrm{k}_1+\mathrm{k}_2-n(\omega)\omega/c}+\frac{n(\omega)-1}{n(\omega)+1}\frac{e^{i(\mathrm{k}_1+\mathrm{k}_2+n(\omega)\omega/c)d}-1}{\mathrm{k}_1+\mathrm{k}_2+n(\omega)\omega/c}\right)\\ &+\mathbf{A}_t(\omega_1)\mathrm{K}^{(2)}(\omega_1,\omega_2)\mathbf{A}_r(\omega_2)\left(\frac{e^{i(\mathrm{k}_1-\mathrm{k}_2-n(\omega)\omega/c)d}-1}{\mathrm{k}_1-\mathrm{k}_2-n(\omega)\omega/c}+\frac{n(\omega)-1}{n(\omega)+1}\frac{e^{i(\mathrm{k}_1-\mathrm{k}_2+n(\omega)\omega/c)d}-1}{\mathrm{k}_1-\mathrm{k}_2+n(\omega)\omega/c}\right)\\ &+\mathbf{A}_r(\omega_1)\mathrm{K}^{(2)}(\omega_1,\omega_2)\mathbf{A}_t(\omega_2)\left(\frac{e^{i(-\mathrm{k}_1+\mathrm{k}_2-n(\omega)\omega/c)d}-1}{-\mathrm{k}_1+\mathrm{k}_2-n(\omega)\omega/c}+\frac{n(\omega)-1}{n(\omega)+1}\frac{e^{i(-\mathrm{k}_1+\mathrm{k}_2+n(\omega)\omega/c)d}-1}{-\mathrm{k}_1+\mathrm{k}_2+n(\omega)\omega/c}\right)\\ &+\mathbf{A}_r(\omega_1)\mathrm{K}^{(2)}(\omega_1,\omega_2)\mathbf{A}_r(\omega_2)\left(\frac{e^{i(-\mathrm{k}_1-\mathrm{k}_2-n(\omega)\omega/c)d}-1}{-\mathrm{k}_1-\mathrm{k}_2-n(\omega)\omega/c}+\frac{n(\omega)-1}{n(\omega)+1}\frac{e^{i(-\mathrm{k}_1-\mathrm{k}_2+n(\omega)\omega/c)d}-1}{-\mathrm{k}_1-\mathrm{k}_2+n(\omega)\omega/c}\right)\Bigg],\end{aligned} \tag{S44}$$

and

$$\mathbf{C}'(\omega) \simeq -\frac{4\pi}{\omega}\frac{1}{n(\omega)+1}\int d\omega_1 d\omega_2\left[\mathbf{A}_t(\omega_1)\mathrm{K}^{(2)}(\omega_1,\omega_2)\mathbf{A}_t(\omega_2)\frac{1}{\mathrm{k}_1+\mathrm{k}_2+n(\omega)\omega/c}\right]\delta(\omega-\omega_1-\omega_2). \tag{S45}$$

The quantities $\mathbf{A}^{[1]}(\omega, d^+) = \mathbf{B}'(\omega)e^{i\omega d/c}$ and $\mathbf{A}^{[1]}(\omega, 0^-) = \mathbf{C}'(\omega)$ are the transmitted and reflected fields respectively that one measures in experiments sensible to the spectral content of the outcoming optical probe pulse, like, e.g., IR-stimulated Raman scattering. Eqs. (S44) and (S45) encode propagation of the pulses within the material as well as the presence of the interface in the TWM case. Notice that when the denominators appearing in these two expressions are minimized, the phase-matching condition is met and the nonlinear response is thus maximized.

SUPPLEMENTARY NOTE 4. THZ PUMP-OPTICAL PROBE EXPERIMENTS

In pump-probe experiments one measures the variations in the transmitted or reflected probe field as function of the time delay t_{pp} between the pump and probe pulses. The transmitted and reflected fields become functions of the time delay, $\mathbf{A}^{[1]}(\omega, t_{pp}, d^+)$ and $\mathbf{A}^{[1]}(\omega, t_{pp}, 0^-)$ respectively, through the external field that appears in $\mathbf{A}_t(\omega, t_{pp})$ and $\mathbf{A}_r(\omega, t_{pp})$,

$$\mathbf{A}^{\mathrm{ext}}(\omega) \to \mathbf{A}^{\mathrm{ext}}(\omega, t_{pp}) = \mathbf{A}_R^{\mathrm{ext}}(\omega) + \mathbf{A}_p^{\mathrm{ext}}(\omega)e^{-i\omega t_{pp}}. \tag{S46}$$

On the contrary, the observation time is fixed $t = t_{\mathrm{gate}}$, and we set $t_{\mathrm{gate}} = 0$ for the sake of simplicity. Fixing the observation time corresponds to an integration over ω in the frequency domain, meaning that the measured quantity in pump-probe experiments e.g. in transmission configuration $\mathbf{A}_{\mathrm{tr}}(t_{pp})$ reads

$$\mathbf{A}_{\mathrm{tr}}(t_{pp}) = \mathbf{A}^{[1]}(t=0, t_{pp}, d^+) = \int \mathbf{A}^{[1]}(\omega, t_{pp}, d^+)d\omega, \tag{S47}$$

while in reflection configuration the measured field $\mathbf{A}_{\mathrm{ref}}(t_{pp})$ reads

$$\mathbf{A}_{\mathrm{ref}}(t_{pp}) = \mathbf{A}^{[1]}(t=0, t_{pp}, 0^-) = \int \mathbf{A}^{[1]}(\omega, t_{pp}, 0^-)d\omega. \tag{S48}$$

The spectral content of the pump-probe response can then be found with a Fourier transform to the frequency ω_{pp}:

$$\mathbf{A}_{\mathrm{tr/ref}}(\omega_{pp}) = \int e^{i\omega_{pp}t_{pp}}\mathbf{A}_{\mathrm{tr/ref}}(t_{pp})dt_{pp}. \tag{S49}$$

Explicitly, the two quantities read

$$
\begin{aligned}
\mathbf{A}_{\rm tr}(\omega_{pp}) = &\int d\omega\ \frac{2\pi}{\omega} t(\omega) f(\omega) e^{i\frac{(n(\omega)+1)\omega d}{c}} \\
\times\Bigg(&\mathbf{A}_t(\omega_{pp}) \mathrm{K}^{(2)}(\omega_{pp}) \mathbf{A}_t(\omega-\omega_{pp}) \left[\frac{e^{i(\mathrm{k}_1+\mathrm{k}_2-n(\omega)\omega/c)d}-1}{\mathrm{k}_1+\mathrm{k}_2-n(\omega)\omega/c} + r(\omega)\frac{e^{i(\mathrm{k}_1+\mathrm{k}_2+n(\omega)\omega/c)d}-1}{\mathrm{k}_1+\mathrm{k}_2+n(\omega)\omega/c} \right] \\
+&\mathbf{A}_t(\omega_{pp}) \mathrm{K}^{(2)}(\omega_{pp}) \mathbf{A}_r(\omega-\omega_{pp}) \left[\frac{e^{i(\mathrm{k}_1-\mathrm{k}_2-n(\omega)\omega/c)d}-1}{\mathrm{k}_1-\mathrm{k}_2-n(\omega)\omega/c} + r(\omega)\frac{e^{i(\mathrm{k}_1-\mathrm{k}_2+n(\omega)\omega/c)d}-1}{\mathrm{k}_1-\mathrm{k}_2+n(\omega)\omega/c} \right] \\
+&\mathbf{A}_r(\omega_{pp}) \mathrm{K}^{(2)}(\omega_{pp}) \mathbf{A}_t(\omega-\omega_{pp}) \left[\frac{e^{i(-\mathrm{k}_1+\mathrm{k}_2-n(\omega)\omega/c)d}-1}{-\mathrm{k}_1+\mathrm{k}_2-n(\omega)\omega/c} + r(\omega)\frac{e^{i(-\mathrm{k}_1+\mathrm{k}_2+n(\omega)\omega/c)d}-1}{-\mathrm{k}_1+\mathrm{k}_2+n(\omega)\omega/c} \right] \\
+&\mathbf{A}_r(\omega_{pp}) \mathrm{K}^{(2)}(\omega_{pp}) \mathbf{A}_r(\omega-\omega_{pp}) \left[\frac{e^{i(-\mathrm{k}_1-\mathrm{k}_2-n(\omega)\omega/c)d}-1}{-\mathrm{k}_1-\mathrm{k}_2-n(\omega)\omega/c} + r(\omega)\frac{e^{i(-\mathrm{k}_1-\mathrm{k}_2+n(\omega)\omega/c)d}-1}{-\mathrm{k}_1-\mathrm{k}_2+n(\omega)\omega/c} \right] \Bigg), \qquad \text{(S50)}
\end{aligned}
$$

which is Eq. (4) in the main text, and

$$
\mathbf{A}_{\rm ref}(\omega_{pp}) = -\int d\omega\ \frac{2\pi}{\omega} t(\omega) \mathbf{A}_t(\omega_{pp}) \mathrm{K}^{(2)}(\omega_{pp}) \mathbf{A}_t(\omega-\omega_{pp}) \left[\frac{1}{\mathrm{k}_1+\mathrm{k}_2+n(\omega)\omega/c} \right], \qquad \text{(S51)}
$$

where now $\mathrm{k}_1 = n(\omega_{pp})\omega_{pp}/c$ and $\mathrm{k}_2 = n(\omega-\omega_{pp})(\omega-\omega_{pp})/c$. In Fig. 3 of the main text we show the quantities $|\mathbf{A}_{\rm tr}(\omega_{pp})|$ and $|\mathbf{A}_{\rm ref}(\omega_{pp})|$. For these plots, we model the spectral content of the incident pulses as Gaussians, $\mathbf{A}_p^{\rm ext}(\omega) = \mathbf{A}_p^0[e^{-(\omega-\omega_p)^2\tau^2/2} - e^{-(\omega+\omega_p)^2\tau^2/2}]$, for the THz pump centered in ω_p and $\mathbf{A}_R^{\rm ext}(\omega) = \mathbf{A}_R^0[e^{-(\omega-\omega_R)^2\tau_R^2/2} - e^{-(\omega+\omega_R)^2\tau_R^2/2}]$ for the probe pulse centered in ω_R, with τ and τ_R controlling their widths and $\mathbf{A}_p^0$ and $\mathbf{A}_R^0$ encoding information on the polarization and intensities of the pulses. The plots in Fig. 3 in the main text are generated having considered narrowband probe pulses ($\omega_R\tau_R \gg 1$) with FWHM equivalent to $0.06\omega_R$. Such choice allows us to identify the phase-matched points along the dispersion curve as the ones that satisfy the phase-matching condition with a continuous-wave laser of frequency ω_R. Moreover, this justifies the assumption we made in the main text that the optical refractive index $n_{\rm eV}$ is constant along the distribution of the optical pulses, although larger pulses may require a more precise modeling of the frequency dependence of the refractive index.

Transmitted signal with realistic broadband THz pump pulses

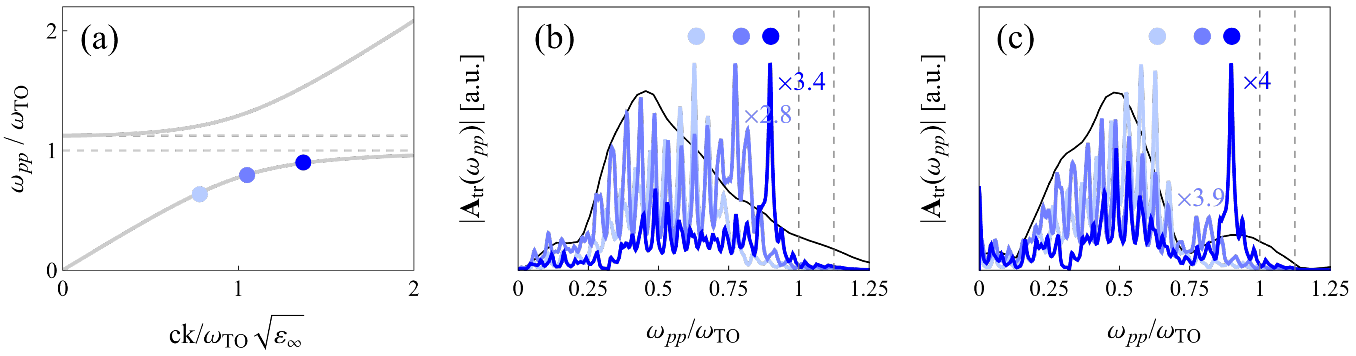

FIG. S1. (a) Phase-matched points in transmission geometry along the phonon polariton dispersion for the spectra of panels (b) and (c), corresponding to the optical refractive index $n_{\rm eV}/\sqrt{\varepsilon_\infty} = 1.20$ (light blue), 1.32 (blue) and 1.54 (dark blue). (a) Transmitted signal $|\mathbf{A}_{\rm tr}(\omega_{pp})|$ induced by a broadband THz pump pulse (black) modeled after a typical DSTMS-generated THz pulse [6]. (b) Transmitted signal $|\mathbf{A}_{\rm tr}(\omega_{pp})|$ induced by a broadband pump pulse (black) modeled after a typical OH1-generated THz pulse [6]. In all panels, $\omega_{\rm LO} = 1.125\omega_{\rm TO}$, $\gamma = 0.01\omega_{\rm TO}$ and $d = 50c/\omega_{\rm TO}\sqrt{\varepsilon_\infty}$. Dashed gray lines highlight $\omega_{\rm TO}$ and $\omega_{\rm LO}$.

When discussing our results for the broadband THz pump, we have considered a Gaussian pulse centered at $\omega_{\rm TO}$, see Fig. 5(d) in the main text, and we have shown that the dominant peak in the nonlinear signal is redshifted with respect to the bare-phonon frequency due to the phase-matching condition being realized along the lower branch of

the PhP dispersion. Present-day high-intensity THz pulses are, however, generated by means of organic emitters, and their spectral content can be significantly different than a simple Gaussian profile, see e.g. Refs. [6–9]. In Fig. S1 we show $|\mathbf{A}_{\rm tr}(\omega_{pp})|$ generated by THz pumps modeled after typical DSTMS (panel b) or OH1 (panel c) THz pulses, with maximum intensity shifted from $\omega_{\rm TO}$. When the pump has sufficiently high intensity on the phase-matched frequency, the latter dominates the response, as expected for a thick sample. A modulation following the pump spectrum is also observed. Instead, if the pump has low intensity on the phase-matched frequency, as one can observe e.g. in Fig. S1(c) for the spectrum obtained with optical refractive index $n_{\rm eV}/\sqrt{\varepsilon_\infty} = 1.32$, the phase-matched peak is suppressed and all the spectral content of the signal resembles that of the pump pulse, modulated by the propagation effects within the material.

We can thus conclude that the dispersion of the PhP can be measured, in the way we discussed in the main text, with THz pulses accessible with present-day technologies. Our comparison between nonlinear signals obtained by DSTMS and OH1 pumps show that the generation crystal should be carefully chosen depending on the range of frequencies that one desires to measure, with broader and featureless pumps preferred.

Effect of sample thickness and penetration depth

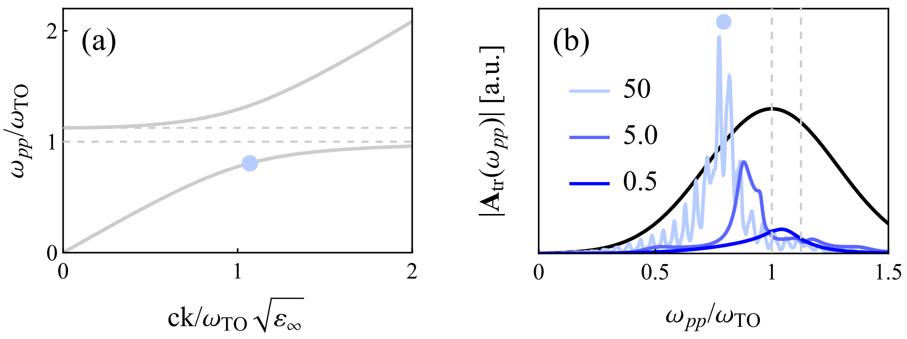

FIG. S2. (a) Phase-matched point in transmission geometry along the phonon polariton dispersion for all spectra of panel (b), corresponding to the optical refractive index $n_{\rm eV}/\sqrt{\varepsilon_\infty} = 1.32$. (b) Transmitted signal $|\mathbf{A}_{\rm tr}(\omega_{pp})|$ for a broadband THz pump (black) and for varying sample thickness d in units of $c/\omega_{\rm TO}\sqrt{\varepsilon_\infty}$ ($\sim \mu$m in typical samples). In the plots, $\omega_{\rm LO} = 1.125\omega_{\rm TO}$ and $\gamma = 0.01\omega_{\rm TO}$. Gray dashed lines highlight $\omega_{\rm TO}$ and $\omega_{\rm LO}$.

In the explicit expression for the transmitted and reflected fields Eqs. (S50) and (S51) the momentum mismatches $\Delta k_\alpha = \sum_i \sigma_i k_i - \alpha n(\omega)\omega/c$ appear, with $\sigma_i = \pm 1$, $i = 1, 2$ and $\alpha = \pm 1$. The square bracket terms in the two expressions contain the relative weight of nonlinear processes with both zero and finite momentum mismatch [10]. As we have considered the case of $d \to +\infty$ in writing Eq. (S51), only the phase-matched process with $k_1 + k_2 + n(\omega)\omega/c = 0$ survives, and it is the only one dominant in reflection geometry. For transmission geometry, however, the weighting factors in Eq. (S50) depend on the sample thickness d and on the penetration depth of the pulses δ through the imaginary part $n''(\omega)$ of the refractive index, i.e. $\delta = c/\omega n''(\omega)$. In samples in which both quantities are much greater than the scale $c/\omega_{\rm TO}\sqrt{\varepsilon_\infty}$ (of the order of μm in typical polar insulators), the weighting factors tend to Dirac-deltas centered on $\Delta k_\alpha = 0$, and phase-matched processes are dominant. One can thus understand why the signal peaks at the phase-matched frequencies, as shown in the plots in the main text. However, when one among d and δ is of the order of $c/\omega_{\rm TO}\sqrt{\varepsilon_\infty}$ or lower, the weighting functions become broad, allowing processes with finite mismatch and thus resulting in the phase-matching condition losing significance. In Fig. S2 we show $|\mathbf{A}_{\rm tr}(\omega_{pp})|$ generated by a broadband THz pulse, having fixed the phase-matched frequency and changing the sample thickness d, with $\delta \gg d$. One can notice that for $d \lesssim c/\omega_{\rm TO}\sqrt{\varepsilon_\infty}$ the Fabry-Perot satellite peaks disappear, as expected, and the frequency of the main peak shifts from the phase-matched one. In this case the spectral content of the outgoing signal comes from the interplay between the spectral weight of $D_0(\omega)$, that mostly resides in $\omega_{\rm TO}$, the transmission coefficient $t(\omega)$ that is minimized at $\omega_{\rm TO}$ but maximized at $\omega_{\rm LO}$, and the spectrum of the pump pulse.

As a last remark, we note that with the phonon polariton dielectric function Eq. (S11) one can get a good estimate of the THz penetration depth, but this expression alone is not in principle sufficient for the optical pulses. In presence of electronic transitions in the eV range, that would strongly affect the penetration depth of the optical pulses, a precise model of the dielectric function should be used.

SUPPLEMENTARY NOTE 5. PROPAGATION EFFECTS ON THE NONLINEAR SIGNAL IN FOUR-WAVE MIXING PROCESSES

The above procedure can be equally well applied to the FWM case with the caveat that the third-order kernel Eq. (S20) is momentum dependent, and thus Eq. (S35) should be rewritten as

$$\mathbf{J}^{(3)}_{[1]}(\omega,z)=\int d\omega_1 d\omega_2 d\omega_3\int_0^d dz'\mathbf{A}^{[0]}(\omega_1,z)\mathbf{A}^{[0]}(\omega_2,z)\mathrm{K}^{(3)}(\omega_1,\omega_2,\omega_3,z-z')\mathbf{A}^{[0]}(\omega_3,z')\delta(\omega-\omega_1-\omega_2-\omega_3),\quad \text{(S52)}$$

where $\mathrm{K}^{(3)}(\omega,z)=\int d\mathrm{k}\ \mathrm{K}^{(3)}(\omega,\mathrm{k})e^{i\mathrm{k}z}$. This Fourier transform can be computed with the residue theorem,

$$\mathrm{K}^{(3)}(\omega,z)=i\mathcal{P}(\omega)\big[\theta(z)e^{in(\omega)\omega z/c}+\theta(-z)e^{-in(\omega)\omega z/c}\big],\quad \text{(S53)}$$

where $\theta(z)$ is the Heaviside step function and $\mathcal{P}(\omega)=\frac{\pi}{\omega^2_{\mathrm{TO}}-\omega^2}\frac{\omega}{c}\frac{\varepsilon(\omega)-\varepsilon_\infty}{\sqrt{\varepsilon(\omega)}}$ is a weighting function reminiscent of the phonon character of the phonon polariton. The integral over z' in Eq. (S52) can be carried out analytically, and the equivalent of Eq. (S38) in the FWM case reads

$$\begin{aligned}\mathbf{J}^{(3)}_{[1]}(\omega,\mathrm{k})&=\int_0^d dz\ \mathbf{J}^{(3)}_{[1]}(\omega,z)e^{-i\mathrm{k}z}\\
&=\int d\omega_1 d\omega_2 d\omega_3\Big[\mathbf{A}_t(\omega_1)\mathbf{A}_t(\omega_2)\mathcal{P}(\omega_{12})\mathbf{A}_t(\omega_3)\mathcal{G}(+1,+1,+1)+\mathbf{A}_t(\omega_1)\mathbf{A}_t(\omega_2)\mathcal{P}(\omega_{12})\mathbf{A}_r(\omega_3)\mathcal{G}(+1,+1,-1)\\
&\qquad+\mathbf{A}_t(\omega_1)\mathbf{A}_r(\omega_2)\mathcal{P}(\omega_{12})\mathbf{A}_t(\omega_3)\mathcal{G}(+1,-1,+1)+\mathbf{A}_t(\omega_1)\mathbf{A}_r(\omega_2)\mathcal{P}(\omega_{12})\mathbf{A}_r(\omega_3)\mathcal{G}(+1,-1,-1)\\
&\qquad+\mathbf{A}_r(\omega_1)\mathbf{A}_t(\omega_2)\mathcal{P}(\omega_{12})\mathbf{A}_t(\omega_3)\mathcal{G}(-1,+1,+1)+\mathbf{A}_r(\omega_1)\mathbf{A}_t(\omega_2)\mathcal{P}(\omega_{12})\mathbf{A}_r(\omega_3)\mathcal{G}(-1,+1,-1)\\
&\qquad+\mathbf{A}_r(\omega_1)\mathbf{A}_r(\omega_2)\mathcal{P}(\omega_{12})\mathbf{A}_t(\omega_3)\mathcal{G}(-1,-1,+1)+\mathbf{A}_r(\omega_1)\mathbf{A}_r(\omega_2)\mathcal{P}(\omega_{12})\mathbf{A}_r(\omega_3)\mathcal{G}(-1,-1,-1)\Big]\\
&\qquad\times\delta(\omega-\omega_1-\omega_2-\omega_3),\end{aligned}\quad \text{(S54)}$$

where $\omega_{12}=\omega_1+\omega_2$, $\mathrm{k}_{12}=n(\omega_{12})\omega_{12}/c$ and

$$\begin{aligned}\mathcal{G}(\alpha_1,\alpha_2,\alpha_3)=&\frac{1}{\mathrm{k}_{12}-\alpha_3\mathrm{k}_3}\left(\frac{1-e^{-i(\mathrm{k}-\alpha_1\mathrm{k}_1-\alpha_2\mathrm{k}_2-\mathrm{k}_{12})d}}{i(\mathrm{k}-\alpha_1\mathrm{k}_1-\alpha_2\mathrm{k}_2-\mathrm{k}_{12})}-\frac{1-e^{-i(\mathrm{k}-\alpha_1\mathrm{k}_1-\alpha_2\mathrm{k}_2-\alpha_3\mathrm{k}_3)d}}{i(\mathrm{k}-\alpha_1\mathrm{k}_1-\alpha_2\mathrm{k}_2-\alpha_3\mathrm{k}_3)}\right)\\
&+\frac{1}{\mathrm{k}_{12}+\alpha_3\mathrm{k}_3}\left(e^{i(\mathrm{k}_{12}+\alpha_3\mathrm{k}_3)d}\frac{1-e^{-i(\mathrm{k}-\alpha_1\mathrm{k}_1-\alpha_2\mathrm{k}_2+\mathrm{k}_{12})d}}{i(\mathrm{k}-\alpha_1\mathrm{k}_1-\alpha_2\mathrm{k}_2+\mathrm{k}_{12})}-\frac{1-e^{-i(\mathrm{k}-\alpha_1\mathrm{k}_1-\alpha\mathrm{k}_2-\alpha_3\mathrm{k}_3)d}}{i(\mathrm{k}-\alpha_1\mathrm{k}_1-\alpha_2\mathrm{k}_2-\alpha_3\mathrm{k}_3)}\right).\end{aligned}\quad \text{(S55)}$$

Eq. (S54) can be used in the unique solution $\mathbf{A}^u(\omega,z)=\frac{4\pi}{c}\frac{1}{2\pi}\int d\mathrm{k}\frac{e^{i\mathrm{k}z}}{\mathrm{k}^2-n^2(\omega)\omega^2/c^2}\mathbf{J}^{(3)}_{[1]}(\omega,\mathrm{k})$ to find the analogous of Eq. (S39). Following the same integration procedure of the TWM case and then using the unique solution in Eqs. (S41) and (S42), that encode the continuity of the fields at the boundaries of the sample, one finds the expressions for the transmitted and reflected fields in the FWM case analogous to Eqs. (S44) and (S45).

For pump-probe experiments like ISRS one should introduce the pump-probe time delay as in the TWM case, that enters through the external field that appears in $\mathbf{A}_t(\omega,t_{pp})$ and $\mathbf{A}_r(\omega,t_{pp})$,

$$\mathbf{A}^{\mathrm{ext}}(\omega)\to\mathbf{A}^{\mathrm{ext}}(\omega,t_{pp})=\mathbf{A}^{\mathrm{ext}}_R(\omega)+\mathbf{A}^{\mathrm{ext}}_R(\omega)e^{-i\omega t_{pp}}.\quad \text{(S56)}$$

As in the TWM case, see Eq. (S46) and the discussion below, the observation time is fixed $t=t_{\mathrm{gate}}=0$ and the spectral content of the pump-probe response $\mathbf{A}_{\mathrm{tr/ref}}(\omega_{pp})$ can be found with a Fourier transform to the frequency ω_{pp}. Because evaluating the transmitted field $|\mathbf{A}_{\mathrm{tr}}(\omega_{pp})|$ in the FWM case is computationally costing, the plots shown in Fig. 2 of the main text are obtained with the TWM expression Eq. (S50) where, instead of $\mathrm{K}^{(2)}(\omega_{pp})$, the weight $\mathcal{P}(\omega_{pp})$ was used. Treating the FWM as an effective-TWM process can be justified by noting that Eq. (S38) and (S54) are formally equivalent, with the weighting factors $\mathcal{G}(\alpha_1,\alpha_2,\alpha_3)$ containing the information on the phase-matching condition that is equivalently contained in the weighting factors of Eq. (S38). This approximation gives qualitatively good results, although for quantitative predictions one should compute $|\mathbf{A}_{\mathrm{tr}}(\omega_{pp})|$ with the proper expression that the procedure outlined above gives.

---

* niccolo.sellati@uniroma1.it

† lara.benfatto@roma1.infn.it
‡ mattia.udina@uniroma1.it

[1] Nagaosa, N. & Heusler, S. *Quantum Field Theory in Condensed Matter Physics* Texts and monographs in physics (Springer, New York, NY, 1999). URL https://books.google.it/books?id=C9uAXYIlFhMC.
[2] Mahan, G. D. *Many Particle Physics, Third Edition* (Plenum, New York, 2000).
[3] Wahlstrand, J. K. & Merlin, R. Cherenkov radiation emitted by ultrafast laser pulses and the generation of coherent polaritons. *Phys. Rev. B* **68**, 054301 (2003). URL https://link.aps.org/doi/10.1103/PhysRevB.68.054301.
[4] Dougherty, T. P., Wiederrecht, G. P. & Nelson, K. A. Impulsive stimulated raman scattering experiments in the polariton regime. *J. Opt. Soc. Am. B* **9**, 2179–2189 (1992). URL https://opg.optica.org/josab/abstract.cfm?URI=josab-9-12-2179.
[5] Fu, Z. & Yamaguchi, M. Coherent excitation of optical phonons in gaas by broadband terahertz pulses. *Scientific Reports* **6**, 38264 (2016). URL https://doi.org/10.1038/srep38264.
[6] Knighton, B. E. *et al.* Terahertz waveform considerations for nonlinearly driving lattice vibrations. *Journal of Applied Physics* **125**, 144101 (2019). URL https://doi.org/10.1063/1.5052638.
[7] Shalaby, M. & Hauri, C. P. Demonstration of a low-frequency three-dimensional terahertz bullet with extreme brightness. *Nature Communications* **6**, 5976 (2015). URL https://doi.org/10.1038/ncomms6976.
[8] Somma, C. *et al.* Ultra-broadband terahertz pulses generated in the organic crystal dstms. *Opt. Lett.* **40**, 3404–3407 (2015). URL https://opg.optica.org/ol/abstract.cfm?URI=ol-40-14-3404.
[9] Dastrup, B. S., Hall, J. R. & Johnson, J. A. Experimental determination of the interatomic potential in LiNbO3 via ultrafast lattice control. *Applied Physics Letters* **110**, 162901 (2017). URL https://doi.org/10.1063/1.4980112.
[10] Mukamel, S. *Principles of Nonlinear Optical Spectroscopy* (Oxford University Press, 1995).
